# Effects of exercise on cardiorespiratory fitness in children and adolescents with overweight and obesity: a systematic review and meta-analysis of 72 randomized controlled trials

**DOI:** 10.1186/s12889-025-25254-y

**Published:** 2025-11-11

**Authors:** Jie Men, Zhengyang Yu, Weiqi An, Peiling Wang, Xinyu Hou, Yuxi Zhang, Simin Wu, Guoyu Zhu, Pengbo Wang, Chenglong Cui, Yu Zhang, Jingwen Wang, Jiaxin Ding, Yaoyong Wang

**Affiliations:** 1 Fenyang College of Shanxi Medical University, Fenyang, 032200 China; 2 Department of Respiratory and Critical Care Medicine, Fenyang Hospital of Shanxi Province, Fenyang, 032200 China; 3 Key discipline of Fenyang College of Shanxi Medical University Physiology, Fenyang, 032200 China; 4 The First Affiliated Hospital of Zhengzhou University, Zhengzhou, 450052 China; 5 Fenyang Hospital Affiliated to Shanxi Medical University, Fenyang, 032200, China

**Keywords:** Cardiorespiratory fitness, Children and adolescents, Overweight or obesity, Sports medicine

## Abstract

**Objective:**

Evaluate the impact of exercise on the cardiorespiratory fitness of the children and adolescents with overweight or obesity, and explore possible influencing factors.

**Study design:**

This meta-analysis adhered to the Preferred Reporting Items for Systematic Reviews and Meta-Analyses (PRISMA) guidelines and included randomized controlled trials (RCTs) published between January 2005 and January 2025. Data were retrieved from PubMed, Embase, Cochrane Library, CNKI, WanFang, and other databases. The included studies evaluated the effects of exercise on six cardiorespiratory fitness (CRF) indicators.

**Results:**

A total of 72 eligible RCTs with 5,320 children and adolescents with overweight or obesity were included (The mean age and BMI of participants were 12.93 ± 2.47 years and 28.08 ± 4.74 kg/m², respectively). Compared to the control group, exercise was associated with improvements in Body Mass Index(MD:-1.14[-1.57,-0.71] kg/m^2^, *P* < 0.00001), maximal oxygen uptake(MD:2.43[1.51,3.34] ml kg⁻¹ min⁻¹, *P* < 0.00001), peak oxygen uptake(MD:2.06[1.12,2.99] ml kg⁻¹ min⁻¹, *P* < 0.00001), systolic blood pressure(MD:-3.16[-5.00,-1.31] mmHg, *P* < 0.00001), diastolic blood pressure(MD:-1.38[-2.13,-0.63] mmHg, *P* = 0.0003), and resting heart rate(MD: -3.23[-4.70,-1.76] bpm, *P* < 0.0001).

**Conclusion and relevation:**

This study demonstrates that exercise is an effective tool for improving low CRF caused by overweight and obesity in children and adolescents. Exercise programs with a duration of ≤ 12 weeks, a frequency of ≥ 3 sessions per week, and a session length of ≤ 60 min were associated with greater improvements in CRF. Combined exercise or moderate-to-high-intensity interval training can achieve superior improvements compared with conventional exercise programs. In addition, stricter supervision plans are necessary during exercise.

**Supplementary Information:**

The online version contains supplementary material available at 10.1186/s12889-025-25254-y.

## Introduction

Evidence suggests that the global decline in cardiorespiratory fitness (CRF) among children and adolescents due to insufficient physical activity has been inevitable over the past decades [1, 2]. Compared to traditional risk factors such as obesity, smoking, type 2 diabetes, hyperlipidemia, and hypertension, CRF is a more direct predictor of mortality risk [3]. Several authoritative guidelines, including those of the American Heart Association [4], and the European Society of Cardiology, have incorporated CRF into clinical evaluations [5]. Studies indicate that for every 1 MET reduction in CRF in the children and adolescents with overweight or obesity, the risk of all-cause mortality and cardiovascular disease (CVD) mortality increases by 13% and 15%, respectively [6]. Notably, 81% of children and adolescents experience insufficient physical activity, combined with a lack of emphasis on CRF, which further exacerbates health threats associated with low CRF [7], Therefore, preventing and improving low CRF caused by overweight and obesity in children and adolescents is crucial.

Insufficient physical activity and poor dietary management are major causes of high BMI and low CRF [8]. However, some studies focusing on children and adolescents with obesity have reported a positive association between BMI and vital capacity [9–11]. This conclusion can be misleading. In reality, this population tends to obtain higher absolute values in vital capacity tests[12], which may be attributed to larger body size interfering with the test results. Nevertheless, this does not necessarily indicate better CRF, and adjusted data have also confirmed this finding [13]. VO_2max_, as the gold standard of CRF, provides an objective reflection of cardiorespiratory health. It comprehensively measures the efficiency of oxygen uptake, transport, and utilization, and is strongly and inversely associated with all-cause mortality [14]. Improvements in CRF through exercise rely on multisystem physiological remodeling. On the one hand, exercise induces physiological cardiac remodeling, enhances pumping efficiency, increases stroke volume, reverses obesity-related compensatory elevations in cardiac output, and optimizes oxygen transport capacity [15, 16]. On the other hand, exercise upregulates PGC-1α expression, promoting skeletal muscle mitochondrial biogenesis [17] (e.g., via the NRF1/TFAM pathway), thereby improving skeletal muscle oxygen utilization. In addition, exercise stimulates endothelial eNOS activity through shear stress of blood flow, increases nitric oxide (NO) bioavailability [18], improves vasodilatory function, and repairs impaired endothelial function and vascular health. Together, these mechanisms provide further evidence supporting the role of exercise in improving CRF. Thus, weight control remains an effective way to improve CRF. Currently, weight control and obesity treatment mainly rely on pharmacological therapies and bariatric surgeries [19], both of which have achieved significant results in improving obesity. However, there is no evidence that these methods significantly improve CRF. On the contrary, patients often experience adverse effects such as decreased exercise capacity, nausea, increased heart rate, and elevated blood pressure after treatment [20], indirectly undermining their CRF levels. Numerous studies have shown that aerobic, resistance, and high-intensity interval training (HIIT), either individually or in combination, effectively improve CRF. Moreover, these findings align with the World Health Organization’s Guidelines on Physical Activity and Sedentary Behavior [21]which recommend 45–60 min of moderate-to-high-intensity physical activity daily for children and adolescents. This further underscores the confidence and importance of promoting exercise to improve CRF, although specific exercise dosage for CRF improvement is not explicitly provided. In addition to improving CRF, exercise can effectively control body weight and improve metabolic and cardiac health [22, 23]. These benefits have been consistently observed across populations of different ages, sexes, and regions, and exercise [24, 25], as a globally popular health intervention, has been widely validated in studies conducted in multiple countries [26].

The consensus that exercise improves CRF is well established, but previous studies still have limitations. First, nearly all studies focus on the effects of specific training methods on individuals with illnesses [27, 28], resulting in a lack of generalizability. Second, some studies have not conducted subgroup analyses on factors that could influence results, such as exercise duration, frequency, and cycle. Additionally, the inclusion scope of participants (e.g., ethnicity and country) is relatively limited [29, 30]. Wang et al.[31] highlighted in their meta-analysis that exercise significantly improves some CRF-related indicators in the children and adolescents with overweight or obesity. However, the study included only a few references, with the most represented CRF outcome indicator, VO_2max_, being based on just five studies. As a result, CRF-related findings in that study are inevitably prone to instability and publication bias due to limited statistical power. This study systematically included nearly all literature on exercise’s impact on CRF in the children and adolescents with overweight or obesity to ensure accuracy and generalizability. It is based on the largest global sample and evaluates factors such as gender, region, and exercise protocols on CRF improvement in this group. This provides critical evidence for exercise dosage recommendations and clinical practice.

## Materials and methods

### Protocol registration

This review followed the Preferred Reporting Items for Systematic Reviews and Meta-Analyses (PRISMA) guidelines [32]. It was registered in the International Prospective Register of Systematic Reviews (PROSPERO) on October 4, 2024 (registration number CRD42024596928).

### Information sources and search strategy

Databases including PubMed, Embase, Cochrane Library, CNKI, and WanFang were searched electronically, with a time frame from 2005 to January 2025.The search strategy was based on the framework of participants, interventions, outcomes, and study design. Mesh terms and free-text words were used, including: (1) exercise, aerobic exercise, resistance training, physical exercise, acute exercise, exercise training, strength training, endurance exercise; (2) adiposity, overweight, obesity, fat; (3) adolescent, children, youngster, teenager, kid; (4) cardiorespiratory fitness, cardiorespiratory endurance, aerobic capacity. It was registered in the International Prospective Register of Systematic Reviews (PROSPERO) on October 4, 2024. The complete search strategy for the databases is provided in Supplementary eTable 1.

### Data extraction and Inclusion/Exclusion criteria

Data extraction included basic information of the included studies (first author’s name, title, year of publication, and study location), participant characteristics (age, sex, sample size), and training variables (intensity, type, frequency, and intervention). The physiological criteria for exercise intensity were defined as follows: moderate-to-low intensity, ≤ 76% HRmax; high intensity, > 76% HRmax or equivalent intensity according to other evaluation standards [33]. In addition, detailed information on the exercise intervention categories and intensity of the included studies has been provided in the supplementary material (see eTable 3. Basic features of the included studies). To calculate effect sizes, baseline and follow-up mean values and standard deviations (Mean ± SDs) were extracted for both intervention and control groups duration), main outcomes, and key elements for bias risk assessment. If the required data were missing, the corresponding authors were contacted for provision. Studies were excluded if relevant data could not be obtained. Studies meeting the following criteria were included: (1) Study type: randomized controlled trials (RCTs).(2) Participants: The children and adolescents with overweight or obesity aged 5 ~ 19 years (BMI ≥ 25), regardless of country, ethnicity, or gender.(3) Interventions: Any form of exercise, including but not limited to aerobic exercise, resistance training, and various intensity levels of physical activity.(4)Main outcome indicator: ①Body Mass index(BMI); ② Maximum oxygen uptake (VO_2max_); ③ Peak oxygen uptake (VO_2peak_); ④ Systolic blood pressure (SBP); ⑤ Diastolic blood pressure (DBP); ⑥ Resting heart rate (HR_rest_); Exploratory outcome indicator: Maximum heart rate (HR_max_). It should be noted that differences in laboratory testing protocols and field testing protocols may lead to variations in the results of VO_2max_ and VO_2peak_ [34].The following types of studies were excluded:① Studies without access to the full text or raw data; ② Duplicate publications; ③ Studies involving athletes as participants.

### Risk of bias assessment, sensitivity analysis and GRADE

The Cochrane Risk of Bias Tool 2.0 was used to assess RCTs [35], which includes three levels: low risk, high risk and unclear risk. It evaluates aspects such as randomization methods, allocation concealment, blinding of participants and personnel, blinding of outcome assessors, completeness of outcome data, selective reporting, and other potential sources of bias. Each domain was rated as low risk, moderate risk, serious risk, critical risk, or no information [36]. Two reviewers (YZY and AWQ) independently assessed bias risk and any disagreements were resolved by a third party (MJ). Additionally, funnel plots were used to assess publication bias when the meta-analysis included ≥ 10 studies. To enhance result stability, sensitivity analyses were conducted by excluding individual studies for the same outcome. We used the GRADE (Grading of Recommendations, Assessment, Development, and Evaluation) approach (reference), and two investigators (YZY and AWQ) independently assessed the certainty of evidence for each outcome. The assessment domains included study limitations (risk of bias), inconsistency, indirectness, imprecision, and publication bias. The quality of evidence was categorized as very low, low, moderate, or high. This analysis has been added to the Methods Sect [37].

### Data synthesis and analysis

Statistical analyses were performed using Review Manager 5.3 (RevMan) software. For continuous data, mean difference (MD) was used as the effect size statistic, and all effect sizes were reported with 95% confidence intervals (CIs).Heterogeneity was assessed using the *I*^*2*^ statistic, with thresholds of 25%, 50%, and 75% indicating low, moderate, and high heterogeneity, respectively [38].

The meta-analysis significance level was set at α = 0.05. When *I*^*2*^ ≤ 25%, a fixed-effects model was used for pooled analysis; when *I*^*2*^ > 25%, a random-effects model was applied; when *I*^*2*^ ≥ 50%, subgroup analyses were conducted to explore the association between individual differences and intervention efficacy, considering factors such as sex, baseline BMI, and region. In addition, subgroup analyses were performed for key moderating factors (e.g., exercise frequency, duration, and cycle) to ensure generalizability and accuracy of the results.

## Results

### Study selection

**Fig. 1:**
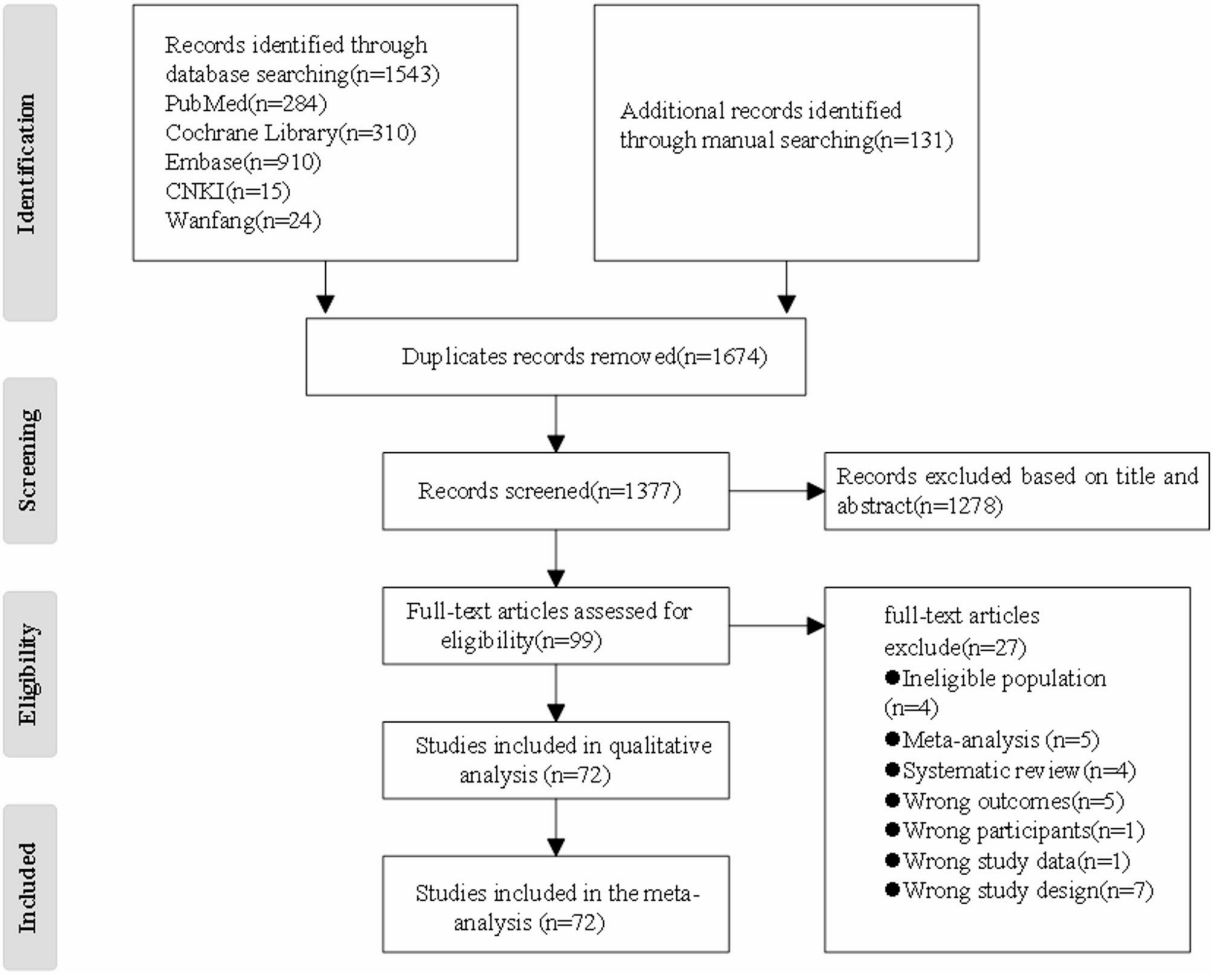
Process flowchart of research selection

**Fig. 2:**
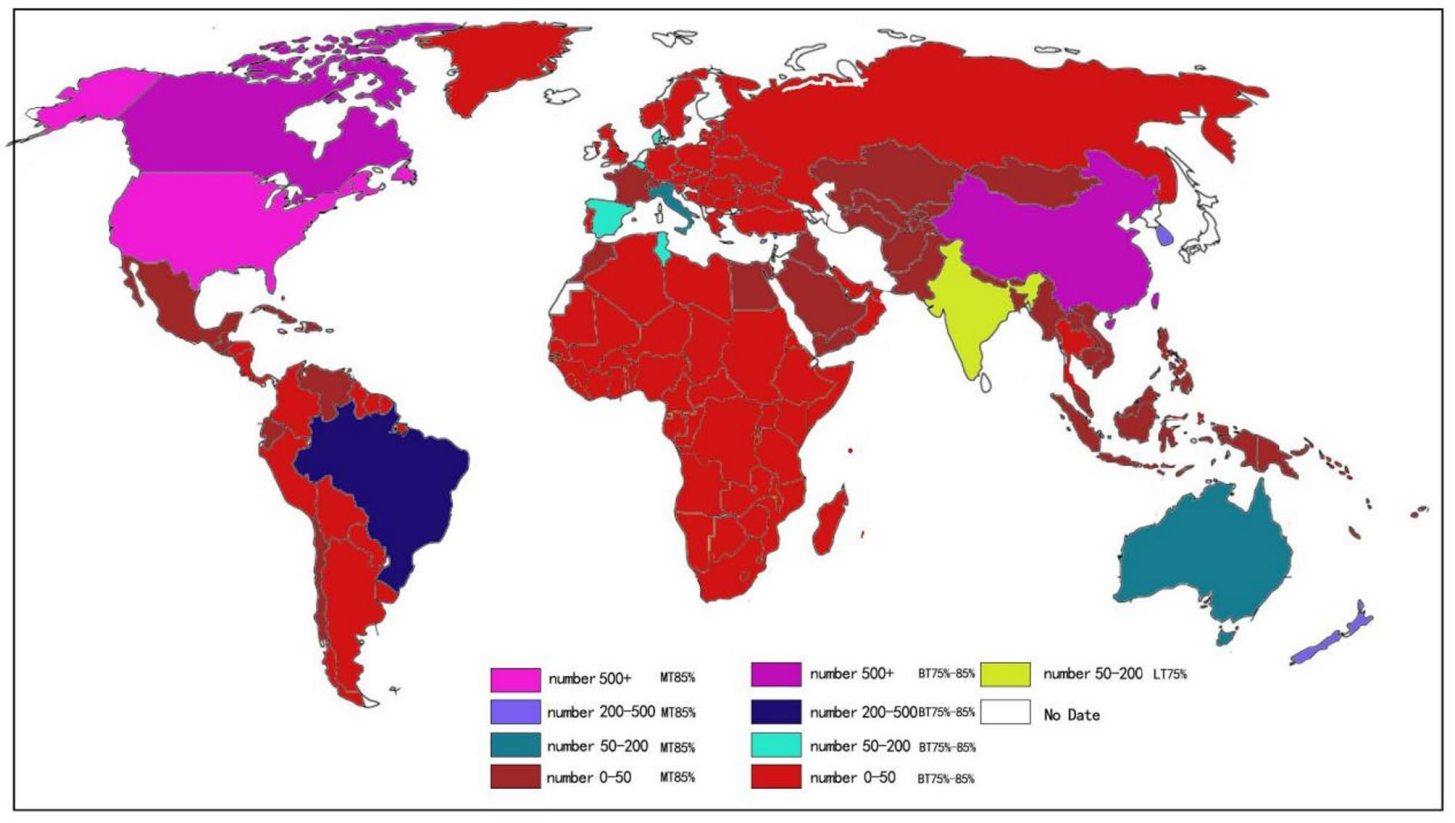
Distribution characteristics of included samples. LT75%: The proportion of sports shortage minors in the region is less than 75%; BT75 ~ 85%: The proportion of minors in the region is between 75 and 85; MT85%: The proportion of sports shortage minors in the region is more than 75%

Figure 1 provides a detailed description of the PRISMA flow. A total of 1562 studies were retrieved from the databases and 131 studies were identified from other sources. After rigorous screening, 72 studies were included, all of which were eligible RCTs involving 5320 children and adolescents with overweight or obesity (The mean age and BMI of participants were 12.93 ± 2.47 years and 28.08 ± 4.74 kg/m², respectively). Basic features of the included studies and excluded studies and reasons for exclusionis is provided in Supplementary eTable 2 and eTable 3. Among them, 3065 participants were in the exercise and 2255 were in the control group, respectively. All studies reported exercise frequency, duration, period, and methods for measuring outcome indicators, 36 of which reported detailed medical supervision, whereas 36 did not. Additionally, 28 studies reported 406 participants (7.6% of the total) withdrawing due to family or personal reasons. Nine adverse events were reported, with an adverse event rate of 0.2% (only one was related to exercise). Figure 2 shows the geographical and sample distribution of the included studies: 18.1% were conducted in Europe, 22.2% in North America, 9.7% in South America, 34.7% in Asia, 8.3% in Australia, and 7% in Africa, covering 25 countries and regions. The sample distribution was as follows: New Zealand: 392, Switzerland: 36, Turkey: 39, Tunis: 173, Thailand: 37, Serbia: 79, China: 1402, USA: 824, Belgium: 91, Korea: 290, Portugal: 40, Iran: 90, Brazil: 286, Italy: 140, Saudi Arabia: 27, Canada: 857, Spain: 98, Australia: 75, Singapore: 24, France: 31, India: 74, Estonia: 42, Germany: 38, UK: 29, Denmark: 106, for a total of 5320 participants.

### Assessment sensitivity analysis, risk of bias, publication bias and GRADE for included studies

The overall risk of bias across the 72 included RCTs was low. Assessments were as follows: random sequence generation, 69/72; allocation concealment, 67/72; blinding of participants and providers, 27/72; blinding of assessors, 28/72; incomplete outcome data, 71/72; selective reporting, 71/72; and other biases, 70/72(For details, please refer to eTable 4). A sensitivity analysis was conducted for all six outcomes by removing individual studies. The pooled effect sizes showed no significant changes, indicating that the results of this study were relatively stable, publication bias was assessed, and funnel plots were largely symmetrical (Risk of bias graph and Funnel plot data for including studies is provided in Supplementary eFigure 1, eFigure 2 and eFigure 3.) indicated no evidence of a publication bias [39]. The certainty of evidence assessed using the GRADE approach was rated as low for VO_2max_ and DBP, and moderate for VO_2peak_, HR_rest_ and SBP. These ratings were primarily attributable to the high heterogeneity and risk of bias observed in some of the included studies. Detailed GRADE assessments are provided in Supplementary eTable 7.

### Heterogeneity analysis

Our meta-analysis revealed substantial heterogeneity for VO_2max_, VO_2peak_, SBP, and DBP, with I² values of 86%, 91%, 97%, and 86%, respectively. However, even after conducting subgroup analyses based on variables such as sex, region, and exercise dose (including frequency, duration, and intervention period), high heterogeneity persisted (I²> 75%). This may be attributable to methodological differences or to other unaccounted factors, including age, pubertal status, baseline levels, and measurement protocols.

### Meta-analysis results

#### Respiratory function

**Fig. 3:**
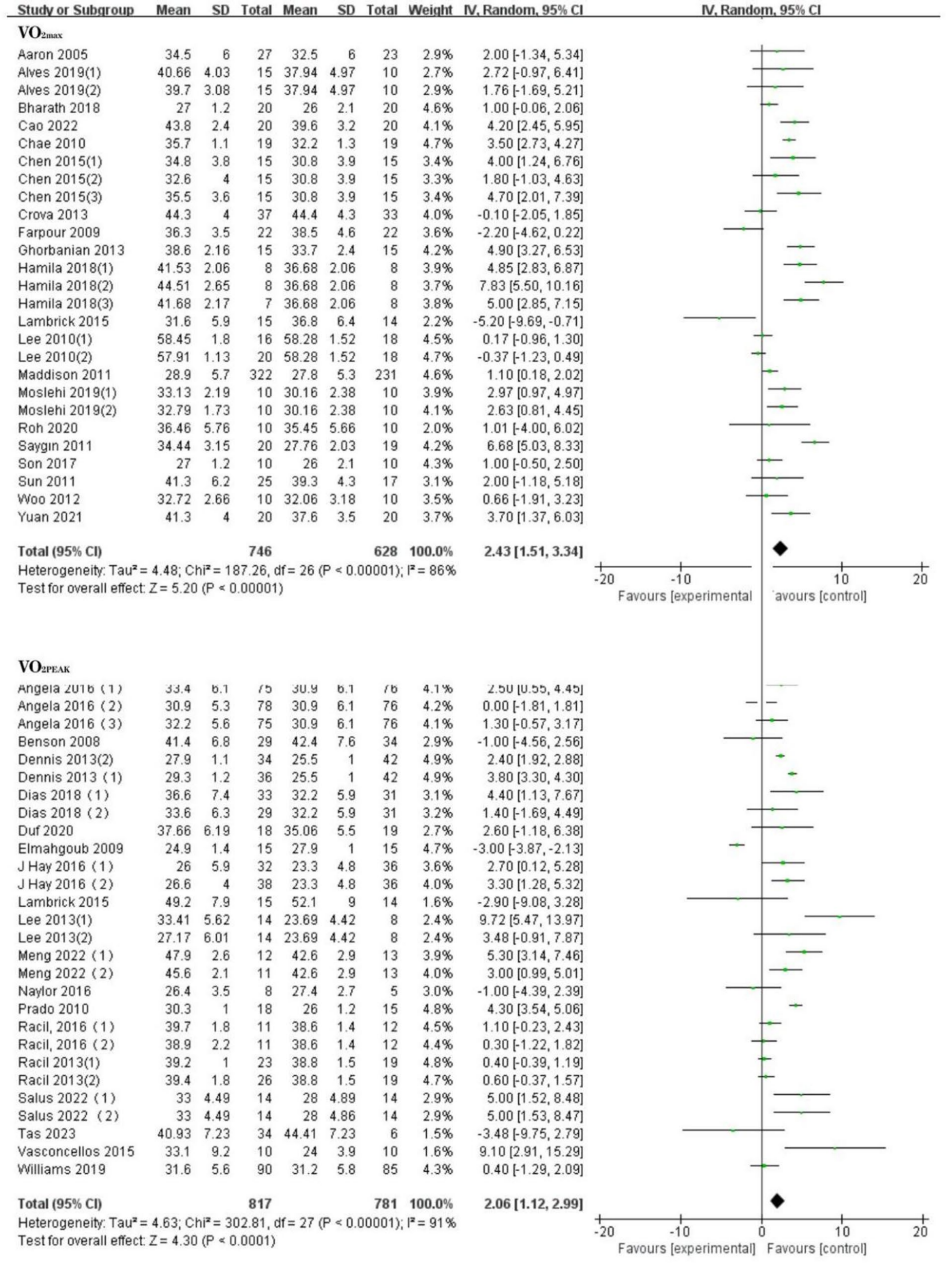
Forest plot of meta-analysis on the effect of VO_2max_ and VO_2peak_

Compared to the control group, exercise was significantly associated with improvements in VO_2max_ and VO_2peak_ among the children and adolescents with overweight or obesity. VO_2max_ included 20 RCTs [40–59](MD:2.43[1.51, 3.34]ml kg^−1^ min^−1^, *I*^2^ = 86%),VO_2peak_ included 19 RCTs [27, 47, 60–76](MD:2.10[1.27, 2.94]ml kg^−1^ min^−1^, *I*^2^ = 88%).(See Fig. 3 for details).

### Cardiovascular function

#### Blood pressure

**Fig. 4:**
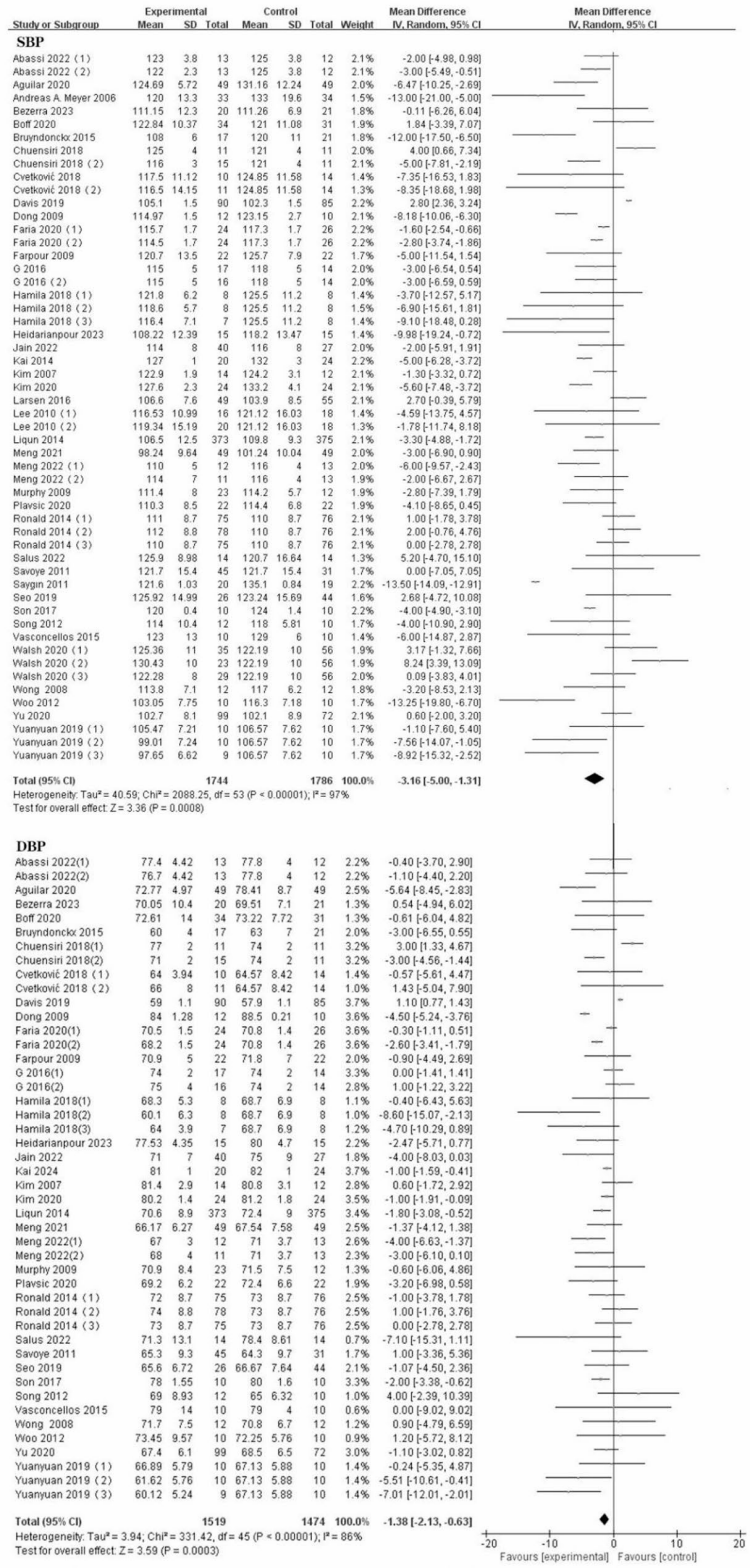
Forest plot of meta-analysis on the effect of SBP and DBP

Compared with the control group, exercise was significantly associated with improvements in SBP and DBP in the children and adolescents with overweight or obesity. SBP included 39 RCTs [28, 29, 33, 36, 38, 39, 44, 47, 50, 60–88] (MD: −2.73[−3.93, −1.52]mm Hg, *I*^*2*^ = 91%), and DBP included 34 RCTs [28, 29, 33, 39, 44, 45, 47, 50, 60, 62–64, 66, 67, 69, 70, 89–99] (MD: −1.38[−2.13, −0.63]mmHg, *I*^*2*^ = 86%). (See Fig. 4 for details)

#### Heart rate

**Fig. 5:**
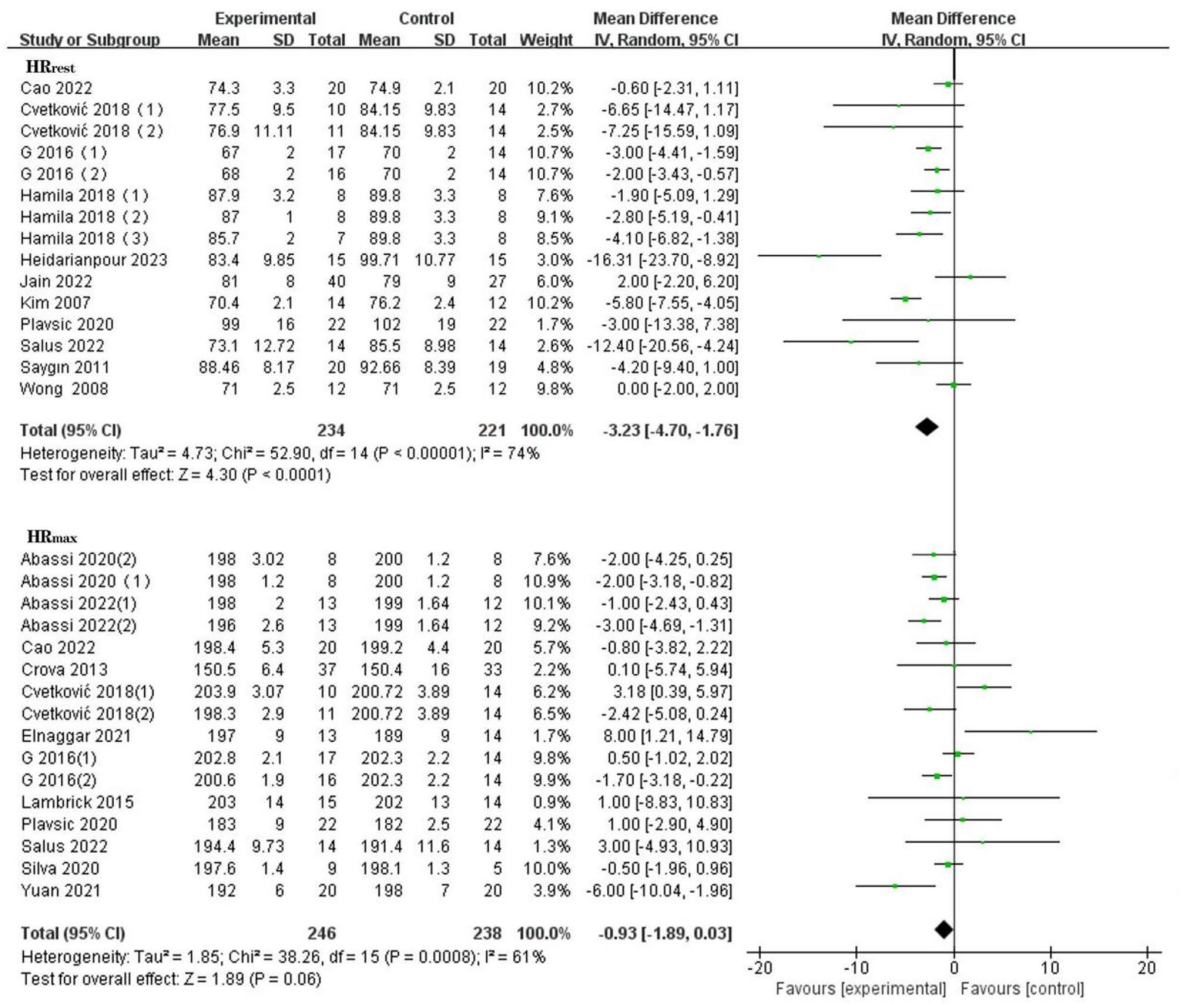
Forest plot of meta-analysis on the effect of HR_rest_ and HR_max_

Compared with the control group, exercise was significantly associated with improvements in HR_rest_, but not HR_max,_ among the children and adolescents with overweight or obesity. HR_rest_ included 12 RCTs [28, 47, 48, 62, 77–84] (MD: −3.34[−4.77, −1.91]), and HR_max_ included 10 RCTs [45, 62, 78–81, 85–88] (MD: −0.93[−1.89, 0.03]; *I*^*2*^ *=* 51%). (See Fig. 5 for detail).

#### Subgroup analysis results

In this study, detailed subgroup analyses were conducted to identify key factors that might cause heterogeneity. The results showed that adjusting exercise duration, frequency, and session length, as well as altering exercise intensity and modality, significantly affected CRF improvements. However, due to the relatively limited studies on different regions and genders, a comprehensive evaluation of these intervention effects was not feasible. Nonetheless, existing data revealed a clear trend in oxygen uptake: exercise interventions had more pronounced effects on improving CRF in the children and adolescents with overweight or obesity from Asia, particularly males. In addition, combined or moderate-to-high-intensity interval exercise was more effective than single exercise modalities. Overall, an exercise duration ≤ 12 weeks, frequency ≥ 3 sessions per week, and session length ≤ 60 min were more effective in significantly improving CRF levels in the children and adolescents with overweight or obesity. These findings further support the effectiveness of exercise as a preventive and therapeutic intervention. Statistical table of subgroup analysis results. Statistical table and figure of subgroup analysis results is provided in Supplementary eTable 5 and eFigure5, eFigure6 and eFigure7.

## Discussion

This meta-analysis included 72 eligible RCTs involving 5320 children and adolescents, making it the largest global evaluation of the impact of exercise on CRF in overweight and obesity youth, with samples encompassing individuals with health conditions such as hypertension, cardiovascular diseases, and diabetes. In this meta-analysis, we found that exercise significantly improved BMI, VO_2max_, VO_2peak_, SBP, DBP, and HR_rest_ in the children and adolescents with overweight or obesity [89–91], which aligns with Martin-Smith’s conclusions [92], providing further evidence for the role of exercise in improving CRF in this population. Similarly, comparable results have also been observed in middle-aged and older adult populations [93, 94]. Subgroup results indicated that different exercise protocols or stratified studies based on participants’ region and baseline physical conditions could achieve varying improvements, including significant enhancements in respiratory and cardiovascular functions.

Although VO_2max_ and VO_2peak_ are both commonly used indicators for assessing CRF, they reflect different aspects. VO_2max_ represents the physiological upper limit of the body’s oxygen delivery system and serves as an objective physiological maximum, whereas VO_2peak_ reflects the level of subjective effort. While VO_2peak_ is not considered the optimal standard for evaluating CRF, it is more frequently used in clinical practice and has been shown to provide equivalent value [95]. Both VO_2max_ and VO_2peak_ are significantly and inversely associated with the risk of various diseases and all-cause mortality [3, 96]. The American Heart Association (AHA) has emphasized that a decline in CRF of 2–3 metabolic equivalents (METs) can increase the risk of coronary heart disease (CHD) and all-cause mortality by 2–5 times. Conversely, each 1-MET increase in CRF can reduce the risk of type 2 diabetes, certain cancers, and all-cause mortality by more than 10%[3, 97]. Notably, a longitudinal study with more than 10 years of follow-up further confirmed that regular exercise can effectively slow the age-related decline in VO_2peak_ [98]. Our results provide evidence supporting the role of exercise in improving oxygen uptake capacity. Subgroup analysis revealed that the improvement in VO_2max_ due to exercise applies to the children and adolescents with overweight or obesity with varying BMI baselines. Among them, improvements were most pronounced in the Asian population. This is closely related to the implementation of various health policies for minors in Asian countries in recent years (e.g., “Healthy China 2030"[99], “Health Japan 21"[100]) under WHO’s global physical activity guidelines, along with stricter oversight in physical education classes. Compared with single exercise modalities, combined exercise or moderate-to-high-intensity interval exercise (≥ 60% VO_2max_ or ≥ 60% HR_max_) was more effective in improving VO_2max_. This may be because, in addition to enhancing aerobic capacity, the anaerobic phases of training induce the activation of signaling pathways such as AMPK, p38 MAPK, and CaMK, which in turn upregulate the expression of peroxisome proliferator–activated receptor gamma PGC-1α[17]. This process promotes mitochondrial biogenesis and function, thereby enhancing oxygen utilization efficiency. The synergistic effect of these two phases provides the biological basis for exercise-induced mitochondrial remodeling that improves VO_2max_, which is particularly critical for enhancing CRF in children and adolescents with overweight or obesity. Additionally, we found that exercise dosage significantly correlates with outcomes, with the most effective improvements in oxygen uptake observed for interventions lasting ≤ 12 weeks, ≤ 3 sessions per week and ≤ 60 min per session. This because extended training durations may lead to overtraining and the “diminishing returns of training adaptation” phenomenon [101, 102]. Therefore, more scientifically structured training regimens are crucial for improving CRF.

Blood pressure (BP) directly reflects vascular function and is closely associated with CRF. During physical activity, CRF is inversely correlated with the extent of BP elevation, which is more pronounced in overweight and obesity youths than in their normal-weight counterparts, reflecting cardiovascular systemic changes mediated by exercise. Hypertension in overweight and obesity minors is a significant risk factor for childhood chronic kidney disease (CKD), adult stroke, and cognitive decline [103]. Moreover, for every 10 mmHg increase in BP, the risk of all-cause mortality and cardiovascular disease (CVD) morbidity in adulthood increases by 12 ~ 15% and 20 ~ 30%, respectively, while those with a high BMI are approximately twice as likely to develop cardiovascular diseases and three times as likely to develop metabolic syndrome in adulthood compared to normal-weight children [104, 105]. The beneficial effects of exercise on blood pressure have been well established. Exercise improves endothelial function and vascular structure by activating the eNOS/NO pathway through vascular shear stress [18]. It can also induce reprogramming of the autonomic nervous system (enhancing parasympathetic activity and suppressing sympathetic activity) [106], thereby optimizing central regulation and improving blood pressure control. In addition, exercise enhances insulin sensitivity, reversing metabolic disturbances and their adverse effects on the vasculature [107]. Together, these mechanisms constitute the physiological basis for the blood pressure–lowering effects of exercise, and our findings further support this view. These mechanisms are the primary reasons why exercise improves BP, and our findings support this conclusion. However, interventions lasting ≤ 12 weeks, with a frequency of ≥ 3 sessions per week and 60 min per session, showed better BP outcomes, aligning with the “moderate, high-frequency” training principle [102]. Additionally, long-duration interventions and comprehensive supervision significantly enhanced the effectiveness of exercise in improving BP; specifically, two six-month exercise interventions reduced SBP by 13 mmHg [108] and 13.5 mmHg [53], respectively, while exercise combined with comprehensive supervision lowered SBP by 12 mmHg [109]. Compared to the exercise-only group, these combined measures improved the outcomes by 311.4%, 319.3%, and 279.7%, respectively. Therefore, future studies should further explore optimizing blood pressure management through long-term and comprehensively monitored exercise training. It should be noted that blood pressure is also influenced by age, developmental stage, and responsiveness to exercise; these factors should be carefully considered when interpreting the effects of exercise on blood pressure.

HR_rest_ is an independent and simple predictor of CVD incidence and mortality. In a 5 ~ 36 year epidemiological study involving 10,000 participants, HR_rest_ was significantly negatively correlated with survival rates in CVD patients [110]; for every 5 bpm increase in HR_rest_, the risk of hospitalization for heart failure increased by 16%, and CVD mortality risk increased by 13%; conversely, every 5 bpm reduction in HR_rest_ resulted in a 12% decrease in all-cause mortality [110]. Several clinical studies support the improvement of HR_rest_ with exercise, which is associated with an increase in stroke volume, regulation of the autonomic nervous system, and improvements in metabolic and hormone levels, all of which are linked to better vascular health, consistent with our findings. Additionally, we found that combined exercise is more effective in improving HR_rest_ than single exercise modalities. In two reports by Marit Salus, Vallo Tillmann, and others, the effect sizes of HR_rest_ improvement were 6.65 bpm, 7.25 bpm, and 12.40 bpm in three groups, which were 99.1%, 117.1%, and 271.3% higher than the overall exercise group effect size [62, 78]; In one study targeting obesity female children [77], combined exercise with a strict supervision plan reduced HR_rest_ by 16.31 bpm, which was 388.3% higher than the total exercise group effect size. Regarding exercise improvement in overweight and obesity individuals. Our study found that exercise had no significant effect on HR_max,_ which can be largely attributed to the fact that HR_max_ is primarily determined by age [111]. Any adaptive changes in response to exercise are minimal, highly variable across individuals [112], and remain a matter of debate within the academic community [113, 114].Therefore, research should instead focus on indicators that more accurately reflect the functional and structural adaptations of the human body induced by training.

Heterogeneity is one of the key factors in evaluating the quality of evidence. In our analysis, we observed high heterogeneity for VO_2max_, VO_2peak_, SBP, and DBP. In addition to addressing this through random-effects models and subgroup analyses, it is also important to consider the influence of age and pubertal development on CRF, which are closely linked to differences in growth and sex hormone levels. Studies have shown that before puberty, increases in cardiac output are primarily achieved through elevated heart rate, whereas after puberty, they rely more heavily on increases in stroke volume. These physiological differences directly contribute to variations in VO_2max_ measurements and in responses to exercise [115]. Measurement protocols and baseline differences are other critical factors to consider. Although laboratory testing is undoubtedly the gold standard, practical constraints related to equipment, physiological demands, and personnel often necessitate field testing, which inevitably introduces measurement error [34]. Baseline levels also serve as an important moderator of intervention effects, as individuals with lower baseline levels typically experience greater relative improvements [116]. Exercise intensity should likewise be considered, though in our analysis we did not observe significant differences in the effects of intensity on VO_2max_ or VO_2peak_, which may be attributable to the limited number of high-intensity studies included. When interpreting the high heterogeneity observed for blood pressure outcomes, factors such as age, pubertal status, testing protocols, baseline levels, exercise intensity, and cultural background should also be taken into account. Physiological differences related to age and developmental stage, non-linear changes in response to exercise associated with baseline levels [116, 117], exercise intensity, and cultural variability are all likely to contribute to heterogeneity. Therefore, these factors should be carefully considered when interpreting the results.

### Limitations

This meta-analysis has several limitations. First, only Chinese and English publications were included, which may have introduced language bias. Second, although the use of adult BMI cutoff values (BMI ≥ 25) as the standard for defining overweight and obesity in children and adolescents is relatively common, it lacks age- and sex-specific considerations and does not adequately capture the dynamic changes associated with growth and development. Therefore, future studies are recommended to adopt the WHO or IOTF age- and sex-specific percentiles to enhance the scientific rigor and accuracy of assessment. Third, most participants in the included studies were recruited from school settings, with limited data available from non-school populations, which may restrict the generalizability of the findings to broader social contexts. Fourth, high heterogeneity posed a major challenge in this meta-analysis, limiting the applicability and generalization of the results. Fifth, although adverse events were rare (9 cases, 0.2%, with only one event directly related to exercise), underreporting or insufficient monitoring in the included studies may have contributed to this outcome; hence, caution should be exercised when interpreting the safety of exercise interventions. Finally, this study primarily focused on evaluating the effects of interventions after the onset of low cardiorespiratory fitness rather than preventive strategies. While interventions remain critical for health management, this emphasis may underestimate the value of prevention in promoting health.

## Conclusion

The results of this meta-analysis indicate that exercise has a positive effect on improving low CRF in the children and adolescents with overweight or obesity. Additionally, We found that exercise programs with a duration of ≤ 12 weeks, a frequency of ≥ 3 sessions per week, and a session length of ≤ 60 min were associated with greater improvements in CRF; however, the practical effects may be influenced by individual differences among participant. Furthermore, combined exercise or moderate-to-high intensity interval training is more effective than single exercise modalities. Also, more stringent monitoring plans help to further enhance the benefits of CRF improvement. These findings provide further evidence for the clinical or family health management of the children and adolescents with overweight or obesity, as well as for the development of relevant exercise guidelines and recommendations.

## Supplementary Information

Supplementary Material 1.

## Data Availability

No datasets were generated or analysed during the current study.

## Abbreviations

CRF: Cardiorespiratory fitness
CVD: Cardiovascular disease
HIIT: High-intensity interval training
RCTs: Randomized controlled trials
VO_2peak_: Peak oxygen uptake
SBP: Systolic blood pressure
DBP: Diastolic blood pressure
HR_rest_: Resting heart rate
HR_max_: Maximum heart rate
VO_2__max_: Maximum oxygen uptake
AHA: The American Heart Association
CHD: Coronary heart disease
CKD: Chronic kidney disease
CVD: Cardiovascular disease
NO: Nitric oxide
