# Supplementary material for: Effects of exercise on cardiorespiratory fitness in children and adolescents with overweight and obesity: a systematic review and meta-analysis of 72 randomized controlled trials
Source: BMC Public Health. 2025 Nov 11;25:3899. doi: 10.1186/s12889-025-25254-y (PMC12607143; doi:10.1186/s12889-025-25254-y)
Supplement: Supplementary file 1 — Supplementary Material 1. [file 12889_2025_25254_MOESM1_ESM.docx]

**Supplemental Online Content**

eTable 1. Search strategy

eTable 2. Excluded studies and reasons for exclusion

eTable 3.Basic features of the included studies

eTable 4. Quality assessment for including studies

eTable 5. Statistical table of subgroup analysis results

eTable 6. Forest plot data for including studies

eTable 7. GRADE summary of findings for 5 outcomes across RCTs.

eFigure 1. Risk of bias graph

eFigure 2. Risk of bias summary

eFigure 3. Funnel plot

eFigure 4. Results of subgroup analysis of maximal oxygen consumption

eFigure 5. Results of subgroup analysis of Peak oxygen consumption

eFigure 6. Results of subgroup analysis of systolic pressure

eFigure 7. Results of subgroup analysis of diastolic pressure

eFigure 8. Results of subgroup analysis of Heart rate max

eFigure 9. Results of subgroup analysis of Heart rate rest

eTable 1. Search strategy

### PubMed

| **Items** | **Search Terms** | **Search Results** |
| --- | --- | --- |
| #1 | adolescent[MeSH Terms] | 2243758 |
| #2 | (((children[Title/Abstract]) OR (youngster[Title/Abstract])) OR (teenager[Title/Abstract])) OR (kid[Title/Abstract]) | 1313494 |
| #3 | adiposity[MeSH Terms] | 15917 |
| #4 | ((overweight[Title/Abstract]) OR (obesity[Title/Abstract])) OR (fat[Title/Abstract]) | 635574 |
| #5 | exercise[MeSH Terms] | 256067 |
| #6 | ((((((mind body exercises[Title/Abstract]) OR (aerobic exercise[Title/Abstract])) OR (resistance training[Title/Abstract])) OR (physical exercise[Title/Abstract])) OR (acute exercise[Title/Abstract])) OR (exercise training[Title/Abstract])) OR (strength training[Title/Abstract] (endurance exercise[Title/Abstract])) | 72790 |
| #7 | cardiorespiratory fitness[MeSH Terms] | 3480 |
| #8 | ((Cardiopulmonary function[Title/Abstract]) OR (Cardiopulmonary endurance[Title/Abstract])) OR (aerobic capacity[Title/Abstract])) | 8712 |
| #9 | #1 or #2 | 3134107 |
| #10 | #3 or #4 | 638624 |
| #11 | #5 or #6 | 289309 |
| #12 | #7 or #8 | 11936 |
| #13 | #9 and #10 and #11 and #12 1984-2024 | 325 |
| #14 | 2005-2024 | 284 |

### Embase

| **Items** | **Search Terms** | **Search Results** |
| --- | --- | --- |
| #1 | 'adolescent'/exp | 2123109 |
| #2 | adolescent:ab,ti OR youngster:ab,ti OR teenager:ab,ti OR kid:ab,ti | 214400 |
| #3 | 'obesity'/exp | 705480 |
| #4 | obesity:ab,ti OR adiposity:ab,ti OR fat:ab,ti OR overweight:ab,ti | 884444 |
| #5 | 'exercise'/exp | 737195 |
| #6 | exercise:ab,ti OR 'mind body exercises':ab,ti OR 'aerobic exercise':ab,ti OR 'resistance training':ab,ti OR 'physical exercise':ab,ti OR 'acute exercise':ab,ti OR 'exercise training':ab,ti OR 'strength training':ab,ti OR 'endurance exercise':ab,ti | 449236 |
| #7 | 'cardiorespiratory fitness'/exp | 13523 |
| #8 | 'cardiorespiratory fitness':ab,ti OR 'Cardiopulmonary function':ab,ti OR 'Cardiopulmonary endurance':ab,ti OR 'cardiorespiratory fitness':ab,ti OR ' | 33005 |
| #9 | #1 OR #2 | 2127471 |
| #10 | #3 OR #4 | 1111164 |
| #11 | #5 OR #6 | 738667 |
| #12 | #7 OR #8 | 36591 |
| #13 | #9 AND #10 AND #11 AND #12 | 964 |
| #14 | #9 AND #10 AND #11 AND #12 AND [2005-2024]/py | 910 |

**Cochrane Library**

| **Items** | **Search Terms** | **Search Results** |
| --- | --- | --- |
| #1 | MeSH descriptor: [Adolescent] explode all trees | 136839 |
| #2 | (children):ti,ab,kw OR (youngster):ti,ab,kw OR (teenager):ti,ab,kw OR (kid):ti,ab,kw | 190465 |
| #3 | MeSH descriptor: [Adiposity] explode all trees | 1123 |
| #4 | (overweight):ti,ab,kw OR (obesity):ti,ab,kw OR (fat):ti,ab,kw | 86119 |
| #5 | MeSH descriptor: [Exercise] explode all trees | 38714 |
| #6 | (mind body exercises):ti,ab,kw OR (aerobic exercise):ti,ab,kw OR (resistance training):ti,ab,kw OR (physical exercise):ti,ab,kw OR (acute exercise):ti,ab,kw | 79860 |
| #7 | (exercise training):ti,ab,kw OR (strength training):ti,ab,kw OR (endurance exercise):ti,ab,kw | 57847 |
| #8 | MeSH descriptor: [cardiorespiratory fitness] explode all trees | 629 |
| #9 | (aerobic capacity):ti,ab,kw OR (Cardiopulmonary function):ti,ab,kw OR (Cardiopulmonary endurance):ti,ab,kw OR (cardiorespiratory fitness):ti,ab,kw | 13555 |
| #10 | #1 or #2 | 284645 |
| #11 | #3 or #4 | 86182 |
| #12 | #5 or #6 or #7 | 108950 |
| #13 | #8 or #9 | 13555 |
| #14 | #10 and #11 and #12 and #13 | 310 |
|  | with Publication Year from 2005 to 2024, in Trials | 308 |

| **Search Terms** | **Search Results** |
| --- | --- |
| (SU="Adolescent" OR SU="children" OR SU="teenager") AND (SU="exercise" OR SU="aerobic exercise" OR SU="physical exercise" OR SU="physical and mental exercise" OR SU="strength training" OR SU="resistance training" OR SU="acute exercise" OR SU="physical training") AND (SU="obesity" OR SU="overweigh") AND (SU="cardiopulmonary function" OR SU="cardiorespiratory fitness" OR SU="aerobic capacity" OR SU="cardiorespiratory endurance") | 15 |

**CNKI**

### WanFang

| **Search Terms** | **Search Results** |
| --- | --- |
| (Theme="Adolescent" or Theme="children" or Theme="teenager") and (Theme="exercise" or Theme="aerobic exercise" or Theme="physical exercise" or Theme="physical and mental exercise" or Theme="strength training" or Theme="resistance training" or Theme="acute exercise" or Theme="physical training") and (Theme="obesity" or Theme="overweigh") and (Theme="cardiopulmonary function" or Theme="cardiorespiratory fitness" or Theme="aerobic capacity" or Theme="cardiorespiratory endurance") | 24 |

eTable 2. Excluded studies and reasons for exclusion

| N |  | Title | Reason for exclusion |
| --- | --- | --- | --- |
| 1 | Tang(2007)^1^ | Aerobic-Exercise Training Improves Ventilatory Effi ciency in Overweight Children | Ineligible study data |
| 2 | Logan(2023)^2^ | The complex associations between adiposity, fitness, mental wellbeing and neurocognitive function after exercise: A randomized crossover trial in preadolescent children | Ineligible study data |
| 3 | Peña(2023)^3^ | Inflammatory Mediators and Type 2 Diabetes Risk Factors before and in Response to Lifestyle Intervention among Latino Adolescents with Obesity | Ineligible study data |
| 4 | Pott(2010)^4^ | Course of Depressive Symptoms in Overweight Youth Participating in a Lifestyle Intervention: Associations With Weight Reduction | Ineligible study data |
| 5 | Xi(2022)^5^ | Effectiveness of exercise on body composition for overweight and obese children and adolescents: a network Meta-analysis | Meta-analysis |
| 6 | Martin-Smith(2020)^6^ | High Intensity Interval Training (HIIT) Improves Cardiorespiratory Fitness (CRF) in Healthy, Overweight and Obese Adolescents: A Systematic Review and Meta-Analysis of Controlled Studies | Meta-analysis |
| 7 | Wu(2023)^7^ | Comparative effectiveness of school-based exercise interventions on physical fitness in children and adolescents: asystematic review and network meta-analysis | Meta-analysis |
| 8 | Ribeiro(2023)^8^ | Reply to: Comment on: “The Benefts of Resistance Training in Obese Adolescents: A Systematic Review and Meta-analysis” | Meta-analysis |
| 9 | Wang(2024)^9^ | Effect of high-intensity interval training and moderate-intensity continuous training on cardiovascular risk factors in adolescents: Systematic review and meta-analysis of randomized controlled trials | Meta-analysis |
| 10 | Edward(2016)^10^ | A systematic review of the relationship between asthma, overweight, and the effects of physical activity in youth | Systematic review |
| 11 | Oliveira(2016)^11^ | Effects of group sports on health-related physical fitness of overweight youth: A systematic review and meta-analysis | Systematic review |
| 12 | Wang(2023)^12^ | Physical activity interventions for cardiopulmonary ftness in obese children and adolescents: a systematic reviewand meta-analysis | Systematic review |
| 13 | Martin-Smith(2020)^13^ | High Intensity Interval Training (HIIT) Improves Cardiorespiratory Fitness (CRF) in Healthy, Overweight and Obese Adolescents: A Systematic Review and Meta-Analysis of Controlled Studies | Systematic review |
| 14 | Abassi(2023)^14^ | Effects of moderate- vs. high-intensity interval training on physical fitness, enjoyment, and affective valence in overweight/obese female adolescents: a pre-/post-test study | Wrong outcomes |
| 15 | Haapala(2024)^15^ | Which indices of cardiorespiratory fitness are more strongly associated with brain health in children with overweight/obesity? | Wrong outcomes |
| 16 | Mora-Gonzalez(2024)^16^ | The effects of an exercise intervention on neuroelectric activity and executive function in children with overweight/obesity: The ActiveBrains randomized controlled trial | Wrong outcomes |
| 17 | Panagiotopoulos(2011)^17^ | The Centre for Healthy Weights—Shapedown BC: A Family-Centered, Multidisciplinary Program that Reduces Weight Gain in Obese Children over the Short-Term | Wrong outcomes |
| 18 | Rodriguez-Ayllon(2023)^18^ | The effects of a 20-week exercise program on blood-circulating biomarkers related to brain health in overweight or obese children: The ActiveBrains project | Wrong outcomes |
| 19 | Dai(2023)^19^ | Adaptations to Optimized Interval Training in Soccer Players: A Comparative Analysis of Standardized Methods for Individualizing Interval Interventions | Wrong participants |
| 20 | Bila(2023)^20^ | Body fat, cardiovascular risk factors and polymorphism in the FTO gene: randomized clinical trial and different  physical exercise for adolescents | Wrong study data |
| 21 | Omar (2010)^21^ | Effect of Individualized Exercise Training Combined with Diet Restriction on Inflammatory Markers and IGF-1/IGFBP-3 in Obese Children | Wrong study design |
| 22 | Boer(2014)^22^ | The influence of sprint interval training on body composition, physical and metabolic fitness in adolescents and young adults with intellectual disability: a randomized controlled trial | Wrong study design |
| 23 | Zhou(2023)^23^ | A school-family blended multi-component physical activity program for Fundamental Motor Skills Promotion Program for Obese Children (FMSPPOC): protocol for a cluster randomized controlled trial | Wrong study design |
| 24 | Marques(2023)^24^ | Effects of 12 Weeks of Family and Individual Multi-Disciplinary Intervention in Overweight and Obese Adolescents under Cardiometabolic Risk Parameters: A Clinical Trial | Wrong study design |
| 25 | Wu(2023)^25^ | Effect of virtual reality-based exercise and physical exercise on adolescents with overweight and obesity: study protocol for a randomised controlled trial | Wrong study design |
| 26 | Neumark-Sztainer(2024)^26^ | Eight weeks of high-intensity interval vs. sprint interval training effects on overweight and obese adolescents carried out during the cool-down period of physical education classes: randomized controlled trial | Wrong study design |
| 27 | Tiara(2024)^27^ | Nurse facilitated 5000 m running at Parkrun improves vulnerable adolescent health in a high deprivation area: A matched pair randomized control trial | Wrong study design |

eTable 3.Basic features of the included studies

| Author | Year | Country | Lesion | Age | | Sample | | Intervention measure | | Intervention timouthse | | Outcome indicator |
| --- | --- | --- | --- | --- | --- | --- | --- | --- | --- | --- | --- | --- |
|  |  |  |  | experimental group | control group | experimental group（male/female） | Control group（male/female） | experimental group | control group | experimental group | control group |  |
| Aaron L^28^ | 2005 | American | Obesity | 12.5 ± 0.5 | 12.5 ± 0.7 | 27（13/14） | 23（13/10） | mode of motion:running  exercise time:45 mins  exercise frequency:five times every two weeks  exercise intensity:maximum heart rate of more than 200 beats/min | mode of motion:daily activities | 36 weeks | 36 weeks | 1 |
| Andreas A^29^ | 2006 | American | Overweight and Obesity | 14.2 ± 1.9 | 14.7 ± 2.2 | 33 | 34 | mode of motion:aerobic training  exercise time:70mins  exercise frequency:three times per week  exercise intensity:moderate strength | mode of motion:daily activities | 24 weeks | 24 weeks | 3 |
| Angela^30^ | 2016 | Canada | Obesity | 15.5±1.4 | 15.6±1.3 | 228 | 76 | mode of motion:cycling and running  exercise time:20-45 mins  exercise frequency:four times per week  exercise intensity:65% HRmax | mode of motion:daily activities | 26 weeks | 26 weeks | 2 |
| Alves^31^ | 2019 | Portugal | Overweight | 14.77±1.49 | 14.7±1.49 | 30 | 10 | mode of motion:aerobic exercises  exercise time:60 mins  exercise frequency:two times per week  exercise intensity:75% VO2max | mode of motion:daily activities | 10 weeks | 10 weeks | 1 |
| Abassi^32^ | 2020 | Tunisia | Obesity | 16.1 ± 1.07 | 16.9±1.64 | 16 (0/16) | 8 (0/8) | mode of motion:running  exercise time:25-35 mins  exercise frequency:three times per week  exercise intensity:100–110% MAS | mode of motion:daily activities | 12 weeks | 12 weeks | 5 |
| Aguilar^33^ | 2020 | Spain | Overweight and Obesity | 10.43±1.35 | 10.4±1.35 | 49 | 49 | mode of motion:aerobic exercise  exercise time:90 mins  exercise frequency:four times per week  exercise intensity:Low to moderate intensity | mode of motion:daily activities | 24 weeks | 24 weeks | 3 |
| Abassi^34^ | 2022 | Germany | Obesity | 16.7±0.20 | 16.7±0.20 | 13 | 12 | mode of motion:running  exercise time:30 mins  exercise frequency:three times per week  exercise intensity:100–110% MAS | mode of motion:school physical education program | 12 weeks | 12 weeks | 3,4 |
| Benson^35^ | 2008 | New Zealand | Overweight | 12.3±1.3 | 12.2±1.3 | 32 | 38 | mode of motion:resistance training  exercise time:60 mins  exercise frequency:two times per week  exercise intensity:80% peakHR | mode of motion:daily activities | 8 weeks | 8 weeks | 2 |
| Bruyndonckx^36^ | 2015 | Belgium | Obesity | 15.4±1.5 | 15.1±1.2 | 33 | 28 | mode of motion:Aerobic exercise  exercise time:40 mins  exercise frequency:three times per week  exercise intensity:moderate strength | mode of motion:daily activities | 40weeks | 40weeks | 3,4 |
| Bharath^37^ | 2018 | America | Obesity | 14.6 ± 1 | 14.8 ± 1 | 20（0/20） | 20（0/20） | mode of motion:aerobic exercise  exercise time:40 mins  exercise frequency:five times per week weekexercise intensity:40%-70%  HRmax | mode of motion:daily activities | 12 weeks | 12 weeks | 1 |
| Boff^38^ | 2020 | Brazil | Obesity | 16.42±1.17 | 16.4±1.01 | 34 | 31 | mode of motion:regular physical exercise  exercise time:30 mins  exercise frequency:one times per week  exercise intensity:moderate strength | mode of motion:daily activities | 12weeks | 12weeks | 3,4 |
| Bezerra^39^ | 2023 | Brazil | Overweight | 7.9 ± 1.0 | 7.9 ± 1.0 | 20 | 21 | mode of motion:vigorous physical activity  exercise time:60 mins  exercise frequency:three times per week  exercise intensity:moderate to vigorous | mode of motion:daily activities | 10 weeks | 10 weeks | 3,4 |
| Chae^40^ | 2010 | Korea | Overweight | 10.4±3.1 | 10.6±3.8 | 19(11/8) | 19(10/9) | mode of motion:Aerobic exercise  exercise time:90 mins  exercise frequency:two times per week  exercise intensity:high strength | mode of motion:daily activities | 12 weeks | 12 weeks | 1 |
| Cheng^41^ | 2012 | China | Overweight | 13-14 | 13-14 | 30 | 30 | mode of motion:Aerobic exercise  exercise time:60-70 mins  exercise frequency:two times per week  exercise intensity:moderate strength | mode of motion:daily activities | 8 weeks | 8 weeks | 1,3,4,6 |
| Crova^42^ | 2013 | Italy | Overweight | 9.6 ± 0.5 | 9.6 ± 0.5 | 37(20/17) | 33(15/18) | mode of motion:tennis-specific training  exercise time:120 mins exercise frequency:one time per week  exercise intensity: average heart rate 150.5 ± 6.4 | mode of motion:daily activities | 21 weeks | 121 weeks | 1 |
| Chen^43^ | 2015 | China | Overweight | 13.9±3.1 | 14.4±3.2 | 45 | 15 | mode of motion:Aerobic exercise  exercise time:55-60 mins  exercise frequency:three times per week  exercise intensity:60%VO2max | mode of motion:daily activities | 8 weeks | 8 weeks | 1 |
| Chuensiri^44^ | 2018 | Thailand | Overweight | 11.0 ± 0.3 | 10.6 ± 0.3 | 26 | 11 | mode of motion:resistance training  exercise time:23 mins  exercise frequency:three times per week  exercise intensity:high strength | mode of motion:daily activities | 12weeks | 12weeks | 3,4 |
| Cvetković^45^ | 2018 | Serbia | Overweight | 11-13 | 11-13 | 21 | 14 | mode of motion:football training  exercise time:60 mins  exercise frequency:two times per week  exercise intensity:high intensity interval training | mode of motion:daily activities | 12 weeks | 12 weeks | 3,4,5,6 |
| Cao^46^ | 2022 | China | Obesity | 11.2 ± 0.7 | 10.9 ± 0.4 | 20 | 20 | mode of motion:HIIT  exercise time:18 mins  exercise frequency:three times per week  exercise intensity:100% of MAS | mode of motion:daily activities | 12weeks | 12weeks | 1,5 |
| Dong^47^ | 2009 | China | Overweight and Obesity | 12.23±0.44 | 12.5±0.25 | 12 | 10 | mode of motion:aerobic exercise  exercise time:70-90 mins  exercise frequency:three-four times per week  exercise intensity:50-70% peakHR | mode of motion:daily activities | 24 weeks | 24 weeks | 3,4 |
| Dennis^48^ | 2013 | America | Overweight | 9.3 ±1.1 | 9.3 ±1.1 | 34 | 42 | mode of motion:aerobic exercise  exercise time:20 mins  exercise frequency:seven times per week  exercise intensity:167±7 beats per minute | mode of motion:daily activities | 13 weeks | 13 weeks | 2 |
| Dias^49^ | 2017 | Australia | Obesity | 12.4 ± 1.9 | 11.8 ± 2.4 | 17 | 21 | mode of motion:treadmill running  exercise time:40 mins  exercise frequency:three times per week  exercise intensity:50%-95%HRmax | mode of motion:daily activities | 12 weeks | 12 weeks | 2 |
| Davis^50^ | 2019 | America | Overweight | 9.6 ± 0.73 | 9.7 ± 0.94 | 90 (23/67) | 85(30/55) | mode of motion:aerobic activities  exercise time:40 mins  exercise frequency:five times per week  exercise intensity:HR161 ± 7 beats/min | mode of motion:daily activities | 32 weeks | 32 weeks | 3,4 |
| Duft^51^ | 2020 | Brazil | Overweight and Obesity | 14.44±1.04 | 14.72±1.07 | 18(9/9) | 19(9/10) | mode of motion:resistance training  exercise time:60 mins  exercise frequency:three times per week  exercise intensity:moderate strength | mode of motion:daily activities | 12 weeks | 12 weeks | 2 |
| Elmahgoub^52^ | 2009 | Belgium | Overweight and Obesity | 17±0.11 | 17±0.11 | 15 | 15 | mode of motion:strength and endurance exercises  exercise time:50 mins  exercise frequency:three times per week  exercise intensity:60-75% HRmax | mode of motion:daily activities | 10weeks | 10weeks | 2 |
| Elnaggar^53^ | 2021 | Saudi Arabia | Obesity | 12.69± 2.53 | 13.36±2.17 | 13 | 14 | mode of motion:aerobic exercise  exercise time:25-60 mins  exercise frequency:three times per week  exercise intensity:45% HRmax | mode of motion:daily activities | 8 weeks | 8weeks | 5 |
| Farpour^54^ | 2009 | Switzerland | Obesity | 9.1±1.4 | 8.8±1.6 | 18 | 18 | mode of motion:aerobic exercise  exercise time:60 mins  exercise frequency:three times per week  exercise intensity:55% - 65%HRmax | mode of motion:daily activities | 12 weeks | 12 weeks | 1,3,4 |
| Faria^55^ | 2020 | Brazil | Overweight | 16.1 ± 1.0 | 16.5 ± 1.0 | 24 (10/14) | 26 (11/15) | mode of motion:MICT(walking/running)  exercise time:22.5 mins  exercise frequency:three times per week  exercise intensity55%-65% VO_2_max | mode of motion:daily activities | 12 weeks | 12 weeks | 3,4 |
| Ghorbanian^56^ | 2013 | Iran | Overweight | 17.35±1.07 | 16.9±1.15 | 15 | 15 | mode of motion:interval endurance rope training  exercise time:40 mins  exercise frequency:four times per week | mode of motion:daily activities | 8weeks | 8weeks | 1 |
| Hamila^57^ | 2018 | France | Overweight and Obesity | 14.5 ± 1.0 | 14.5 ± 0.9 | 7(4/3) | 8(5/3) | mode of motion:walking  exercise time:55 mins exercise frequency:three times per week exercise intensity: HR 149 ± 10 bpm | mode of motion:daily activities | 8 weeks | 8 weeks | 1,3,4,6 |
| Heidarianpour^58^ | 2023 | Iran | Obesity | 8.51 ± 0.44 | 8.51±0.44 | 15 | 15 | mode of motion:aerobic and resistance training  exercise time:60 mins  exercise frequency:three times per week  exercise intensity: 55%–65% VO_2_max | mode of motion:daily activities | 12 weeks | 12 weeks | 3,4,6 |
| J Hay^59^ | 2016 | Canada | Overweight | 15.3±1.7 | 15.2±1.7 | 74 | 32 | mode of motion:Self recommended sports  exercise time:30-45mins  exercise frequency:three times per week  exercise intensity:70-85% heart rate | mode of motion:daily activities | 24 weeks | 24 weeks | 2 |
| Jain^60^ | 2022 | Indian | Overweight | 11.7±1.7 | 11.4±1.8 | 46 | 28 | mode of motion:yoga  exercise time:60 mins  exercise frequency:five times per week  exercise intensity:moderate-to-vigorous | mode of motion:daily activities | 18weeks | 18 weeks | 3,4,6 |
| Kim^61^ | 2007 | Korea | Overweight | 17±0.11 | 17±0.11 | 14(14/0) | 12(12/0) | mode of motion:jump rope exercise training  exercise time:40 mins  exercise frequency:three times per week  exercise intensity:high intensity | mode of motion:daily activities | 24 weeks | 24 weeks | 3,4,6 |
| Kim^62^ | 2019 | America | Overweight | 15 ± 1 | 15 ± 1 | 24 (0/24) | 24 (0/24) | mode of motion:jump rope exercise  exercise time:50 mins  exercise frequency:five times per week  exercise intensity:50-60%HRR | mode of motion:daily activities | 12 weeks | 12 weeks | 3,4 |
| Kai^63^ | 2024 | China | Obesity | 14.8±0.8 | 15.0±0.8 | 20 | 24 | mode of motion:Combination movement  exercise time:50 mins  exercise frequency:five times per week  exercise intensity:40%~70%HRR | mode of motion:daily activities | 12 weeks | 12 weeks | 3,4 |
| Lee^64^ | 2010 | Korea | Obesity | 12-14 | 12-14 | 34 | 20 | mode of motion: aerobic exercise  exercise time:60 mins  exercise frequency:three times per week  exercise intensity:70-90%HRmax | mode of motion:daily activities | 10 weeks | 10 weeks | 1,3 |
| Lee^65^ | 2012 | America | Obesity | 15.2 ±1.9 | 14.8±1.4 | 31 | 11 | mode of motion:Aerobic exercise  exercise time:60 mins  exercise frequency:three times per week  exercise intensity:50-75%VO2peak | mode of motion:daily activities | 12 weeks | 12 weeks | 2 |
| Lee^66^ | 2013 | America | Obesity | 14.6 ± 1.9 | 15.0 ± 2.2 | 14(0/14) | 14(0/14) | mode of motion:running  exercise time:60 mins  exercise frequency:three times per week  exercise intensity:60 –75% of VO2peak | mode of motion:daily activities | 12 weeks | 12 weeks | 2 |
| Liqun^67^ | 2014 | China | Overweight | 14.8 ± 1.9 | 14.8 ± 1.9 | 373 | 375 | mode of motion:Students take two classes per day  exercise time:20 mins  exercise frequency:one times per week  exercise intensity:moderate strength | mode of motion:daily activities | 48 weeks | 48 weeks | 3,4 |
| Lambrick^68^ | 2015 | UK | Overweight | 9.3 ± 0.8 | 9.4 ± 0.8 | 15(10/5) | 14(7/7) | mode of motion:daily activities  exercise time:60 mins  exercise frequency:two times per week  exercise intensity:moderate to vigorous | mode of motion:daily activities | 6 weeks | 6 weeks | 1,2,5 |
| Larsen^69^ | 2016 | Denmark | Overweight | 12±0.4 | 12±0.4 | 55 | 51 | mode of motion:Fun Sports  exercise time:60 mins  exercise frequency:seven times per week  exercise intensity:high intensity | mode of motion:daily activities | 6 weeks | 6 weeks | 3 |
| Murphy^70^ | 2009 | America | Overweight and Obesity | 10.21±1.67 | 10.2±1.67 | 23 | 12 | mode of motion:Aerobic exercise  exercise time:30 mins  exercise frequency:five times per week  exercise intensity:moderate strength | mode of motion:daily activities | 12 weeks | 12 weeks | 3,4 |
| Maddison^71^ | 2011 | New Zealand | Overweight | 11.6 ± 1.1 | 11.6 ± 1.1 | 160(116/44) | 162(119/43) | mode of motion:active video games  exercise time:60 mins  exercise frequency:multiple times per week  exercise intensity:moderate to vigorous | mode of motion:daily activities | 24 weeks | 24 weeks | 1 |
| Moslehi^72^ | 2019 | Iran | Overweight and Obesity | 11.12±0.51 | 10.93±0.53 | 10 | 10 | mode of motion:outdoor aerobic exercise  exercise time:25-40 mins  exercise frequency:three times per week  exercise intensity:65%HR-85%HR | mode of motion:daily activities | 8 weeks | 8 weeks | 1 |
| Meng^73^ | 2021 | China | Overweight and Obesity | 16.18±0.79 | 15.36±0.68 | 49 | 49 | mode of motion:Aerobic exercise  exercise time:30 mins  exercise frequency:3-6 times per week  exercise intensity:moderate strength | mode of motion:daily activities | 48 weeks | 48 weeks | 3,4 |
| Meng^74^ | 2022 | China | Overweight | 11.4 ± 0.8 | 11.0 ± 0.7 | 23 | 13 | mode of motion:HIIT  exercise time:11 mins  exercise frequency:three times per week  exercise intensity:90–100% of MAS | mode of motion:daily activities | 12 weeks | 12 weeks | 2,3,4 |
| Naylor^75^ | 2016 | Australia | Obesity | 17.3 ± 0.8 | 15.3 ± 0.8 | 8 | 5 | Mode of motion:Aerobic+Resistance  exercise time:60mins  exercise frequency:three times per week  exercise intensity:65-85%HRmax | mode of motion:daily activities | 12weeks | 12weeks | 2 |
| Prado^76^ | 2010 | Brazil | Overweight and Obesity | 10.6 ± 0.2 | 10.4 ± 0.3 | 18 | 15 | mode of motion:Aerobic exercise  exercise time:60 mins  exercise frequency:three times per week  exercise intensity:moderate strength | mode of motion: normal daily routine | 16weeks | 16weeks | 2 |
| Racil^77^ | 2013 | Tunisia | Obesity | 15.6 ± 0.7 | 15.9 ± 1.2 | 11 | 12 | mode of motion:running  exercise time:40mins  exercise frequency:four times per week  exercise intensity:100 - 110 % MAS | mode of motion:daily activities | 12weeks | 12weeks | 2 |
| Ronald^78^ | 2014 | Canada | Obesity | 15.5±1.4 | 15.6±1.3 | 75（22/53） | 76（22/54） | mode of motion:Aerobic exercise  exercise time:20-45 mins  exercise frequency:four times per week  exercise intensity: 65% -85% HRmax | mode of motion:daily activities | 24weeks | 24weeks | 3,4 |
| Racil^79^ | 2016 | Tunis | Obesity | 14.2±1.2 | 14.2±1.2 | 33 | 14 | mode of motion:jogging ,stretching exercises  exercise time:30 mins  exercise frequency:three times per week  exercise intensity:100%/50% MAS | mode of motion:daily activities | 12weeks | 12weeks | 3,4,5,6 |
| Racial^80^ | 2016 | Tunisia | Obesity | 16.6 ± 0.9 | 6.9 ± 1.0 | 23 | 19 | mode of motion:running  exercise time:30 mins  exercise frequency:6-8 times per week  exercise intensity: 50%-100%VO2peak | mode of motion:daily activities | 12weeks | 12weeks | 2 |
| Roh^81^ | 2020 | Korea | Overweight | 12.60 ± 0.52 | 12.50 ± 0.53 | 10 (7/3) | 10 (7/3) | mode of motion:Taekwondo training  exercise time:60 mins  exercise frequency:five times per week  exercise intensity:moderate strength | mode of motion:daily activities | 16weeks | 16weeks | 1 |
| Saygın^82^ | 2011 | Turkey | Overweight | 10-12 | 10-12 | 20 | 19 | mode of motion:Aerobic exercise  exercise time:60-90 mins  exercise frequency:three times per week  exercise intensity: 50% - 60%HR | mode of motion:daily activities | 12weeks | 12weeks | 1,3,6 |
| Sun^83^ | 2011 | China | Overweight | 13.6±0.7 | 13.6±0.7 | 25(18/7) | 17(7/10) | mode of motion:Aerobic exercise  exercise time:60 mins  exercise frequency:four times per week  exercise intensity: 65% - 80% HRmax | mode of motion:daily activities | 48weeks | 48weeks | 3,4 |
| Savoye^84^ | 2011 | America | Obesity | 12.0 ± 2.5 | 12.5 ± 2.3 | 105(47/58) | 69(22/47) | mode of motion:Aerobic exercise  exercise time:50 mins  exercise frequency:two times per week  exercise intensity:moderate to vigorous | mode of motion:daily activities | 24weeks | 24weeks | 1 |
| Song^85^ | 2012 | Korean | Overweight | 12.7±0.2 | 12.6±0.2 | 12 | 10 | mode of motion:Aerobic exercise  exercise time:50 mins  exercise frequency:three times per week  exercise intensity: 60-70%HRmax | mode of motion:daily activities | 12weeks | 12weeks | 3,4 |
| Silva^86^ | 2012 | Brazil | Obesity | 13-17 | 13-17 | 9 | 5 | mode of motion:Aerobic exercise  exercise time:30-40 mins  exercise frequency:three times per week  exercise intensity:85%HRmax | mode of motion:daily activities | 12weeks | 12weeks | 5 |
| Son^87^ | 2017 | Korean | Obesity | 15±1 | 15±1 | 20（0/20） | 20（0/20） | mode of motion:Aerobic exercise  exercise time:60 mins  exercise frequency:three times per week  exercise intensity:40%-60% HRmax | mode of motion:daily activities | 12weeks | 12weeks | 1,3,4 |
| Seo^88^ | 2019 | Korean | Overweight and Obesity | 12.80 ± 1.72 | 12.09 ± 2.20 | 26 | 44 | mode of motion:walking  exercise time:60 mins  exercise frequency:three times per week  exercise intensity:60%-90%HRmax | mode of motion:daily activities | 16weeks | 16weeks | 3,4 |
| Salus^89^ | 2022 | Estonia | Obesity | 13.1 ± 0.3 | 13.7 ± 0.4 | 14 | 14 | mode of motion:cycling  exercise time:28 mins  exercise frequency:three times per week  exercise intensity:moderate to vigorous | mode of motion:daily activities | 12weeks | 12weeks | 2,3 |
| Salus^90^ | 2022 | Estonia | Obesity | 13.1 ± 1.3 | 13.7 ± 1.6 | 14 | 14 | mode of motion:cycling  exercise time:28 mins  exercise frequency:three times per week  exercise intensity:moderate to vigorous | mode of motion:daily activities | 12weeks | 12weeks | 2 |
| Tas^91^ | 2023 | America | Obesity | 15.2 ± 1.5 | 15.4 ± 1.0 | 31 | 6 | mode of motion:HIIT  exercise time:45 mins  exercise frequency:three times per week  exercise intensity:80% t- 90% HRmax | mode of motion:daily activities | 4weeks | 4weeks | 2 |
| Vasconcellos^92^ | 2015 | Brazil | Obesity | 14.1 ± 1.1 | 14.1 ± 1.1 | 10 | 10 | mode of motion:Aerobic exercise  exercise time:60 mins  exercise frequency:three times per week  exercise intensity:moderate strength | mode of motion:daily activities | 12weeks | 12weeks | 3,4 |
| Wong^93^ | 2008 | Singapore | Obesity | 13.75 ± 1.06 | 14.25 ± 1.54 | 12(12/0) | 12(12/0) | mode of motion:Aerobic exercise  exercise time:40-60 mins  exercise frequency:two times per week  exercise intensity: 65% - 85% HRmax | mode of motion:daily activities | 12weeks | 12weeks | 3,4,6 |
| Woo^94^ | 2012 | Korean | Overweight | 11.30±1.17 | 11.30±1.17 | 10 | 10 | mode of motion:Aerobic exercise  exercise time:60 mins  exercise frequency:regular exercise  exercise intensity: 45-65%HRR | mode of motion:daily activities | 12weeks | 12weeks | 2 |
| Williams^95^ | 2019 | America | Overweight | 10.4 ± 0.87 | 10.4 ± 0.89 | 70 | 67 | mode of motion:Aerobic exercise  exercise time:40 mins  exercise frequency:regular exercise  exercise intensity:moderate strength | mode of motion:daily activities | 32weeks | 32weeks | 2 |
| Walsh^96^ | 2020 | Canada | Obesity | 15.6 ± 1.2 | 15.5 ± 1.3 | 35(12/23) | 56(18/38) | mode of motion:Aerobic exercise  exercise time:20-45 mins  exercise frequency:four times per week  exercise intensity: 65% - 85% HRmax | mode of motion:daily activities | 24weeks | 24weeks | 3 |
| Yuanyuan^97^ | 2019 | china | Overweight | 11.14±1.28 | 11.13±1.26 | 10(6/4) | 10(6/4) | mode of motion:football exercise  exercise time:50 mins  exercise frequency:three times per week  exercise intensity: 54%-64%HRmax | mode of motion:daily activities | 16weeks | 16weeks | 3,4 |
| Yu^98^ | 2020 | China | Overweight | 9.9 ± 0.7 | 9.7 ± 0.6 | 99 (82/17) | 72 (54/18) | mode of motion:Aerobic exercise  exercise time:60 mins  exercise frequency:five times per week  exercise intensity:moderate strength | mode of motion:daily activities | 12weeks | 12weeks | 3,4 |
| Yuan^99^ | 2021 | china | Overweight | 16.1±1.2 | 15.9±1.2 | 20 | 20 | mode of motion:HIIT  exercise time:30-60 mins  exercise frequency:three times per week  exercise intensity: 50%-110%MAP | mode of motion:daily activities | 12weeks | 12weeks | 1,5 |

Notes:1.VO_2max;2._VO_2peak_:3.SBP;4.DBP;5.HRmax；6.HRrest

VO_2max_:maximum oxygen consumption; VO_2peak_:Peak Oxygen Uptake; SBP:systolic blood pressure;DBP:diastolic blood pressure;HR_max_: maximal heart rate; HRrest:resting Heart Rate

eTable 4. Quality assessment for including studies

| Included studies | Year | Random sequence  generation | Allocation concealment | Blinding of participants and providers | Blinding of assessors | Incomplete outcome data | Selective reporting | Other bias |
| --- | --- | --- | --- | --- | --- | --- | --- | --- |
| Aaron L^28^ | 2005 | Low risk | Low risk | High risk | Low risk | Low risk | Low risk | Low risk |
| Andreas A ^29^ | 2006 | Low risk | Low risk | Low risk | High risk | Low risk | Low risk | Low risk |
| Angela^30^ | 2016 | Low risk | Low risk | Unclear | Low risk | Low risk | Low risk | Low risk |
| Alves^31^ | 2019 | Low risk | Low risk | High risk | High risk | Unclear | Low risk | Low risk |
| Abassi^32^ | 2020 | Low risk | Low risk | High risk | High risk | Low risk | Low risk | Low risk |
| Aguilar^33^ | 2020 | Low risk | Low risk | Unclear | Low risk | Low risk | Low risk | Low risk |
| Abassi^34^ | 2022 | Low risk | Low risk | Unclear | Low risk | Low risk | Low risk | Low risk |
| Benson^35^ | 2008 | Low risk | Low risk | High risk | High risk | Low risk | Low risk | Low risk |
| Bruyndonckx^36^ | 2015 | Low risk | Low risk | High risk | High risk | Low risk | Low risk | Low risk |
| Bharath^37^ | 2018 | Low risk | Low risk | Unclear | Low risk | Low risk | Low risk | Low risk |
| Boff^38^ | 2020 | Low risk | Low risk | High risk | Low risk | Low risk | Low risk | Low risk |
| Bezerra^39^ | 2023 | Low risk | Low risk | High risk | High risk | Low risk | Low risk | Low risk |
| Chae^40^ | 2010 | Low risk | High risk | Unclear | Low risk | Low risk | Low risk | Low risk |
| Cheng^41^ | 2012 | Low risk | Low risk | Low risk | High risk | Low risk | Low risk | Low risk |
| Crova^42^ | 2013 | Low risk | Low risk | High risk | High risk | Low risk | Low risk | Low risk |
| Chen^43^ | 2015 | Low risk | Low risk | High risk | Low risk | Low risk | Low risk | Low risk |
| Chuensiri^44^ | 2018 | Low risk | Low risk | High risk | High risk | Low risk | Low risk | Low risk |
| Cvetković^45^ | 2018 | Low risk | Low risk | Low risk | Unclear | Low risk | Low risk | Low risk |
| Cao^46^ | 2022 | Low risk | Low risk | High risk | High risk | Low risk | Low risk | Low risk |
| Dong^47^ | 2009 | Low risk | Low risk | High risk | Low risk | Low risk | Low risk | Low risk |
| Dennis^48^ | 2013 | Unclear | Low risk | Low risk | Low risk | Low risk | Low risk | Low risk |
| Dias^49^ | 2017 | Low risk | Low risk | Low risk | High risk | Low risk | Low risk | Low risk |
| Davis^50^ | 2019 | Low risk | Low risk | Unclear | Unclear | Low risk | Low risk | Low risk |
| Duft^51^ | 2020 | Low risk | Low risk | High risk | Low risk | Low risk | Low risk | Low risk |
| Elmahgoub^52^ | 2009 | Low risk | Low risk | High risk | High risk | Low risk | Low risk | Low risk |
| Elnaggar^53^ | 2021 | Low risk | Low risk | Unclear | Unclear | Low risk | Low risk | Low risk |
| Farpour^54^ | 2009 | Low risk | Low risk | High risk | Low risk | Low risk | Low risk | Low risk |
| Faria^55^ | 2020 | Low risk | Low risk | Low risk | High risk | Low risk | Low risk | Low risk |
| Ghorbanian^56^ | 2013 | Low risk | Low risk | High risk | High risk | Low risk | Low risk | Low risk |
| Hamila^57^ | 2018 | Low risk | Low risk | High risk | High risk | Low risk | Low risk | Low risk |
| Heidarianpour^58^ | 2023 | Low risk | Low risk | Low risk | Low risk | Low risk | Low risk | Low risk |
| J Hay^59^ | 2016 | Low risk | Low risk | Low risk | Unclear | Low risk | Low risk | Low risk |
| Jain^60^ | 2022 | Low risk | Low risk | High risk | High risk | Low risk | Low risk | Low risk |
| Kim^61^ | 2007 | Unclear | Unclear | Low risk | Low risk | Low risk | Low risk | Low risk |
| Kim^62^ | 2019 | Low risk | Low risk | High risk | Unclear | Low risk | Low risk | Low risk |
| Kai^63^ | 2024 | Low risk | Low risk | Low risk | Low risk | Low risk | Low risk | Unclear |
| Lee^64^ | 2010 | Low risk | Unclear | High risk | High risk | Low risk | Low risk | Low risk |
| Lee^65^ | 2012 | Low risk | Low risk | Low risk | Low risk | Low risk | Low risk | Low risk |
| Lee^66^ | 2013 | Low risk | Low risk | Low risk | Unclear | Low risk | Low risk | Low risk |
| Liqun ^67^ | 2014 | Low risk | Low risk | High risk | High risk | Low risk | Low risk | Low risk |
| Lambrick^68^ | 2015 | Low risk | Low risk | Low risk | Low risk | Low risk | Low risk | Low risk |
| Larsen^69^ | 2016 | Low risk | Low risk | Low risk | Unclear | Low risk | Low risk | Low risk |
| Murphy^70^ | 2009 | Low risk | Low risk | High risk | High risk | Low risk | Low risk | Low risk |
| Maddison^71^ | 2011 | Low risk | Low risk | Low risk | Low risk | Low risk | Low risk | Low risk |
| Moslehi^72^ | 2019 | Low risk | Unclear | High risk | High risk | Low risk | Low risk | Low risk |
| Meng ^73^ | 2021 | Low risk | Low risk | Low risk | Low risk | Low risk | Low risk | Low risk |
| Meng^74^ | 2022 | Low risk | Low risk | Low risk | Unclear | Low risk | Low risk | Low risk |
| Naylor^75^ | 2016 | Low risk | Low risk | Low risk | Low risk | Low risk | Low risk | Low risk |
| Prado^76^ | 2010 | Low risk | Low risk | High risk | High risk | Low risk | Low risk | Low risk |
| Racil^77^ | 2013 | Unclear | Low risk | Low risk | Unclear | Low risk | Low risk | Low risk |
| Ronald^78^ | 2014 | Low risk | Low risk | Low risk | Low risk | Low risk | Low risk | Low risk |
| Racil^79^ | 2016 | Low risk | Low risk | Unclear | High risk | Low risk | Low risk | Low risk |
| Racia^80^ | 2016 | Low risk | Low risk | Low risk | Unclear | Low risk | Low risk | Low risk |
| Roh^81^ | 2020 | Low risk | Low risk | Unclear | Low risk | Low risk | Low risk | Low risk |
| Saygın^82^ | 2011 | Low risk | Low risk | Low risk | Unclear | Low risk | Low risk | Low risk |
| Sun^83^ | 2011 | Low risk | Low risk | High risk | High risk | Low risk | Low risk | Unclear |
| Savoye^84^ | 2011 | Low risk | Low risk | High risk | Low risk | Low risk | Low risk | Low risk |
| Song^85^ | 2012 | Low risk | Low risk | Unclear | Unclear | Low risk | Low risk | Low risk |
| Silva^86^ | 2012 | Low risk | Low risk | Unclear | Low risk | Low risk | Low risk | Low risk |
| Son^87^ | 2017 | Low risk | High risk | Low risk | Unclear | Low risk | Low risk | Low risk |
| Seo^88^ | 2019 | Low risk | Low risk | High risk | High risk | Low risk | Low risk | Low risk |
| Salus^89^ | 2022 | Low risk | Low risk | Low risk | Low risk | Low risk | Low risk | Low risk |
| Salus^90^ | 2022 | Low risk | Low risk | High risk | Low risk | Low risk | Low risk | Low risk |
| Tas^90^ | 2023 | Low risk | Low risk | Low risk | High risk | Low risk | Low risk | Low risk |
| Vasconcellos^92^ | 2015 | Low risk | Low risk | Low risk | High risk | Low risk | Low risk | Low risk |
| Wong^93^ | 2008 | Low risk | Low risk | High risk | Low risk | Low risk | Low risk | Low risk |
| Woo^94^ | 2012 | Low risk | Low risk | High risk | Unclear | Low risk | Low risk | Low risk |
| Williams^95^ | 2019 | Low risk | Low risk | Unclear | High risk | Low risk | Low risk | Low risk |
| Walsh^96^ | 2020 | Low risk | Low risk | Low risk | Low risk | Low risk | Unclear | Low risk |
| Yuanyuan^97^ | 2019 | Low risk | Low risk | High risk | High risk | Low risk | Low risk | Low risk |
| Yu^98^ | 2020 | Low risk | Low risk | High risk | Unclear | Low risk | Low risk | Low risk |
| Yuan^99^ | 2021 | Low risk | Low risk | High risk | Unclear | Low risk | Low risk | Low risk |

eTable 5. Statistical table of subgroup analysis results

| Outcomes | Subgroup |  | The Num of studies | MD[95%CI] | *I*²（%） | P value | P Test for subgroup differences |
| --- | --- | --- | --- | --- | --- | --- | --- |
| VO2max | Exercise intensityMotion time | Medium and low  strength | 17 | 2.78 [1.83，3.74] | 82 | <0.00001 | 0.007 |
|  |  | High strength | 3 | 0.55 [-0.77, 1.87] | 71 | 0.41 |  |
|  | Training volume (weeks) | ≤12weeks | 16 | 2.65 [1.62,3.69] | 88 | <0.00001 | 0.01 |
|  |  | ＞12weeks | 4 | 0.95 [0.15,1.75] | 0 | 0.02 |  |
|  | Training volume (weeks) | ≥12weeks | 11 | 1.94 [1.51, 2.37] | 80 | <0.00001 | 0.31 |
|  |  | <12weeks | 9 | 2.27 [1.81, 2.73] | 89 | <0.00001 |  |
|  | Area | Aisa | 13 | 3.24 [2.12,4.36] | 87 | <0.00001 | 0.003 |
|  |  | Europe | 4 | -0.49 [-2.70,1.73 | 63 | 0.67 |  |
|  |  | North America | 2 | 1.09 [0.08,2.10] | 0 | 0.03 |  |
|  |  | Oceania | 1 | 1.10[0.18,2.02] |  | 0.02 |  |
|  | Gender | Male | 3 | 4.30 [3.45, 5.15] | 66 | <0.00001 | <0.00001 |
|  |  | Female | 2 | 1.00 [0.13, 1.87] | 0 | 0.02 |  |
|  | The size of the effect after intervention | High impact of intervention | 4 | 5.38 [4.35,6.42] | 40 | <0.00001 | <0.00001 |
|  |  | Low impact of intervention | 14 | 2.07 [1.25,2.88] | 70 | <0.00001 |  |
|  |  | Fall short of expectations | 3 | -1.83 [-4.12,0.47] | 86 | <0.00001 |  |
|  | motion frequency | ≤three times per week | 16 | 2.43 [1.41,3.45] | 87 | <0.00001 | 0.99 |
|  |  | >three times per week | 4 | 2.41 [-0.05,4.87] | 81 | 0.05 |  |
|  | motion frequency | ≥three times per week | 14 | 1.90 [1.54, 2.27] | 88 | <0.00001 | 0.04 |
|  |  | ＜three times per week | 8 | 2.65 [2.03, 3.27] | 74 | <0.00001 |  |
|  | motion frequency | >three times per week | 3 | 1.65 [1.01, 2.29] | 89 | <0.00001 | 0.08 |
|  |  | =three times per week | 11 | 2.03 [1.59, 2.47] | 89 | <0.00001 |  |
|  |  | <three times per week | 8 | 2.65 [2.03, 3.27] | 74 | <0.00001 |  |
|  | Motion time | ≥50 mins | 14 | 1.50 [1.15, 1.86] | 86 | <0.00001 | <0.00001 |
|  |  | ＜50 mins | 6 | 4.29 [3.61, 4.97] | 53 | <0.00001 |  |
|  | Motion time | ≥60 mins | 12 | 1.40 [1.04, 1.76] | 88 | <0.00001 | <0.00001 |
|  |  | ＜60 mins | 8 | 4.13 [3.51, 4.75] | 44 | <0.00001 |  |

| Outcomes | Subgroup |  | The Num of studies | MD[95%CI] | I²（%） | P value | P Test for subgroup differences |
| --- | --- | --- | --- | --- | --- | --- | --- |
| VO2peak | Gender | Male | 2 | 4.31 [3.05, 5.57] | 0 | <0.00001 | 0.17 |
|  |  | Female | 3 | 2.66 [0.69, 4.63] | 93 | 0.008 |  |
|  | Exercise intensity | Medium and low  strength | 16 | 2.16 [1.10,3.23] | 87 | <0.00001 | 0.80 |
|  |  | High strength | 4 | 2.37 [1.27,2.94] | 86 | <0.00001 |  |
|  | Training volume (weeks) | ≤12weeks | 14 | 1.80 [0.84,2.77] | 79 | 0.00002 | 0.10 |
|  |  | ＞12weeks | 4 | 2.10 [1.27,2.94] | 86 | <0.00001 |  |
|  | Training volume (weeks) | ≥12weeks | 13 | 2.62 [2.36, 2.87] | 86 | <0.00001 | <0.00001 |
|  |  | <12weeks | 5 | -1.37 [-2.03, -0.71] | 85 | <0.0001 |  |
|  | motion frequency | ≤three times per week | 14 | 2.65 [1.15,4.15] | 88 | 0.0005 | 0.1 |
|  |  | >three times per week | 4 | 1.43 [0.40,2.47] | 91 | 0.007 |  |
|  | motion frequency | ≥three times per week | 16 | 2.42 [2.17, 2.66] | 87 | <0.00001 | 0.01 |
|  |  | ＜three times per week | 2 | -1.47 [-4.56, 1.61] | 0 | 0.35 |  |
|  | motion frequency | >three times per week | 4 | 2.26 [1.97, 2.54] | 93 | <0.00001 | 0.005 |
|  |  | =three times per week | 12 | 2.86 [2.39, 3.33] | 83 | <0.00001 |  |
|  |  | <three times per week | 2 | -1.47 [-4.56, 1.61] | 0 | 0.35 |  |
|  | Motion time | ≥50 mins | 8 | 2.91 [2.28, 3.55] | 91 | <0.00001 | 0.08 |
|  |  | ＜50 mins | 10 | 2.30 [2.04, 2.57] | 84 | <0.00001 |  |
|  | Motion time | ≥60 mins | 7 | 3.92 [3.23, 4.61] | 78 | <0.00001 | <0.00001 |
|  |  | <60mins | 11 | 2.18 [1.92, 2.44] | 87 | <0.00001 |  |

| Outcomes | Subgroup |  | The Num of studies | MD[95%CI] | *I*²（%） | P value | P Test for subgroup differences |
| --- | --- | --- | --- | --- | --- | --- | --- |
| SBP | Gender | Male | 8 | -3.54 [-4.41, -2.66] | 71 | <0.00001 | 0.43 |
|  |  | Female | 5 | -3.99 [-4.70, -3.27] | 18 | <0.00001 |  |
|  | Training volume (weeks) | ≤12weeks | 24 | -3.06 [-4.01, -2.12] | 93 | 0.08 | 0.38 |
|  |  | ＞12weeks | 14 | -1.98 [-4.22, 0.27] | 91 | <0.00001 |  |
|  | Training volume (weeks) | ≥12weeks | 33 | -0.66 [-0.95, -0.38] | 93 | <0.00001 | 0.85 |
|  |  | <12weeks | 5 | -0.81 [-2.33, 0.70] | 38 | 0.29 |  |
|  | Exercise intensity | Medium and low  strength | 38 | -2.65 [-3.87, -1.44] | 92 | <0.0001 | 0.31 |
|  |  | High strength | 3 | -6.24 [-13.10, 0.61] | 0 | 0.07 |  |
|  | The size of the effect after intervention | High impact of intervention | 7 | -4.64 [-5.29,-3.98] | 0 | <0.00001 | <0.00001 |
|  |  | Low impact of intervention | 20 | -1.40 [-1.87,-0.93] | 32 | <0.00001 |  |
|  |  | Fall short of expectations | 13 | 1.05 [0.76,1.35] | 0 | <0.00001 |  |
|  | motion frequency | ≤three times per week | 26 | -3.00 [-3.46, -2.54] | 60 | <0.00001 | <0.00001 |
|  |  | >three times per week | 14 | 0.75 [0.39, 1.10] | 95 | <0.0001 |  |
|  | motion frequency | ≥three times per week | 32 | -0.26 [-0.57, 0.05] | 92 | 0.1 | <0.00001 |
|  |  | ＜three times per week | 7 | -2.33 [-2.97, -1.69] | 60 | <0.00001 |  |
|  | motion frequency | >three times per week | 14 | 0.75 [0.39, 1.10] | 95 | <0.0001 | <0.00001 |
|  |  | =three times per week | 17 | -3.72 [-4.38, -3.06] | 55 | <0.00001 |  |
|  |  | <three times per week | 7 | -2.33 [-2.97, -1.69] | 60 | <0.00001 |  |
|  | Motion time | ≥50 mins | 23 | -8.81 [-9.21, -8.40] | 86 | <0.00001 | <0.00001 |
|  |  | ＜50 mins | 15 | 0.63 [0.30, 0.95] | 92 | 0.0002 |  |
|  | Motion time | ≥60 mins | 17 | -4.36 [-5.04, -3.69] | 91 | <0.00001 | <0.00001 |
|  |  | <60mins | 21 | 0.05 [-0.26, 0.36] | 92 | 0.74 |  |
| Outcomes | Subgroup |  | The Num of studies | MD[95%CI] | I²（%） | P value | P Test for subgroup differences |
| DBP | motion frequency | ≤three times per week | 22 | -1.37 [-2.22,-0.53] | 63 | 0.001 | 0.97 |
|  |  | >three times per week | 12 | -1.34 [-2.67,0.02] | 94 | 0.05 |  |
|  | motion frequency | ≥three times per week | 27 | -0.40 [-0.62, -0.17] | 88 | 0.0005 | 0.001 |
|  |  | ＜three times per week | 7 | -1.37 [-1.92, -0.82] | 60 | <0.00001 |  |
|  | motion frequency | >three times per week | 11 | -0.26 [-0.50, -0.02] | 94 | 0.03 | <0.0001 |
|  |  | =three times per week | 15 | -1.16 [-1.73, -0.58] | 66 | <0.0001 |  |
|  |  | <three times per week | 7 | -1.37 [-1.92, -0.82] | 60 | <0.00001 |  |
|  | Motion time | ≥50 mins | 21 | -2.07 [-2.43, -1.71] | 72 | <0.00001 | <0.00001 |
|  |  | ＜50 mins | 13 | 0.22 [-0.03, 0.48] | 86 | 0.08 |  |
|  | Motion time | ≥60 mins | 15 | -3.25 [-3.78, -2.71] | 63 | <0.00001 | <0.00001 |
|  |  | <60mins | 19 | -0.07 [-0.29, 0.16] | 84 | 0.56 |  |
|  | Training volume (weeks) | ≤12weeks | 22 | -1.05 [-1.74,-0.36] | 62 | 0.003 | 0.34 |
|  |  | ＞12weeks | 12 | -1.94 [-3.62,0.26] | 94 | 0.02 |  |
|  | Training volume (weeks) | ≥12weeks | 31 | -1.26 [-2.07, -0.45] | 87 | 0.002 | 0.70 |
|  |  | <12weeks | 3 | -1.94 [-5.23, 1.35] | 56 | 0.25 |  |
|  | Gender | Male | 8 | -0.98 [-1.47, -0.50] | 77 | <0.0001 | 0.82 |
|  |  | Female | 5 | -0.89 [-1.50, -0.29] | 24 | 0.004 |  |
|  | The size of the effect after intervention | High impact of intervention | 18 | -7.93 [-10.42,-5.44] | 95 | <0.00001 | <0.00001 |
|  |  | Low impact of intervention | 14 | -2.08 [-3.25,-2.34] | 0 | <0.00001 |  |
|  |  | Fall short of expectations | 10 | 2.25 [1.38,3.11] | 20 | <0.00001 |  |

| Outcomes | Subgroup |  | The Num of studies | MD[95%CI] | *I*²（%） | P value | P Test for subgroup differences |
| --- | --- | --- | --- | --- | --- | --- | --- |
| HRmax | Exercise intensity | Medium and low  strength | 10 | -0.75 [-2.13,0.63] | 73 | 0.29 | 0.43 |
|  |  | High strength | 3 | 0.22 [-1.73,2.17] | 72 | 0.83 |  |
|  | Training volume (weeks) | ≥12weeks | 9 | -1.23 [-1.76, -0.69] | 61 | <0.00001 | 0.04 |
|  |  | <12weeks | 3 | 3.04 [-1.00, 7.08] | 37 | 0.14 |  |
|  | Gender | Male | 2 | -4.15 [-7.75, -0.55] | 93 | 0.77 | 0.99 |
|  |  | Female | 2 | -1.33 [-2.07, -0.58] | 59 | 0.04 |  |
|  | motion frequency | ＜three times per week | 4 | 0.39 [-1.24, 2.02] | 51 | 0.64 | 0.09 |
|  |  | =three times per week | 8 | -1.07 [-1.61, -0.54] | 75 | <0.0001 |  |
|  | Motion time | ≥50 mins | 4 | 0.39 [-1.24, 2.02] | 51 | 0.64 | <0.0001 |
|  |  | ＜50 mins | 8 | -1.33 [-1.88, -0.78] | 62 | <0.00001 |  |
|  | Motion time | ≥60 mins | 4 | 0.39 [-1.24, 2.02] | 51 | 0.64 | 0.09 |
|  |  | <60mins | 8 | -1.33 [-1.88, -0.78] | 75 | <0.0001 |  |

| Outcomes | Subgroup |  | The Num of studies | MD[95%CI] | *I*²（%） | P value | P Test for subgroup differences |
| --- | --- | --- | --- | --- | --- | --- | --- |
| HRrest | Training volume (weeks) | ≤12weeks | 8 | -3.13 [-4.59, -1.66] | 68 | <0.0001 | 0.04 |
|  |  | ＞12weeks | 2 | 1.30 [-2.60, 5.19] | 0 | 0.51 |  |
|  | Training volume (weeks) | ≥12weeks | 8 | -2.55 [-3.25, -1.85] | 82 | <0.00001 | 0.50 |
|  |  | <12weeks | 3 | -3.11 [-4.59, -1.62] | 0 | <0.0001 |  |
|  | motion frequency | ≤three times per week | 9 | -3.65 [-5.06, -2.23]] | 62 | <0.00001 | 0.01 |
|  |  | >three times per week | 1 | 2.00 [-2.20, 6.20] |  | 0.35 |  |
|  | motion frequency | ≥three times per week | 6 | -2.20 [-3.03, -1.37] | 79 | <0.00001 | 0.06 |
|  |  | ＜three times per week | 4 | -3.70 [-5.00, -2.40] | 0 | <0.00001 |  |
|  | motion frequency | >three times per week | 1 | 2.00 [-2.20, 6.20] |  | 0.35 | 0.02 |
|  |  | =three times per week | 5 | -2.37 [-3.22, -1.53] | 80 | <0.00001 |  |
|  |  | <three times per week | 4 | -3.70 [-5.00, -2.40] | 0 | <0.00001 |  |
|  | Motion time | ≥50 mins | 7 | -3.60 [-4.79, -2.41] | 60 | <0.00001 | 0.05 |
|  |  | ＜50 mins | 3 | -2.13 [-3.00, -1.27] | 72 | <0.000001 |  |
|  | Motion time | ≥60 mins | 4 | -4.43 [-6.39, -2.47] | 75 | <0.00001 | 0.05 |
|  |  | ＜60 mins | 5 | -2.38 [-3.13, -1.63] | 47 | <0.00001 |  |
|  | Gender | Male | 3 | -3.93 [-5.80, -2.05] | 44 | <0.0001 | 0.79 |
|  |  | Female | 2 | -4.43 [-7.73, -1.13] | 86 | 0.008 |  |

eTable 6. Forest plot data for including studies

| Outcomes | The number of studies | *P* | *I*²(%) | effect model | 95%CI |
| --- | --- | --- | --- | --- | --- |
|  |  |  |  |  |  |
| VO2max | 20 | ＜0.00001 | 86 | RE | 2.43 [1.51, 3.34] |
| VO2peak | 19 | <0.00001 | 91 | RE | 2.06 [1.12, 2.99] |
| SBP | 39 | 0.0008 | 97 | RE | -3.16 [-5.00, -1.31] |
| DBP | 34 | 0.0003 | 86 | RE | -1.38 [-2.13, -0.63] |
| HRmax | 12 | 0.06 | 61 | RE | -0.93 [-1.89, 0.03] |
| HRrest | 11 | <0.00001 | 74 | RE | -3.23 [-4.70, -1.76] |

eTable 7. GRADE summary of findings for 5 outcomes across RCTs.

| **Outcome** | **Initial rating** | **Risk of bias** | **Inconsistency** | **Indirectness** | **Imprecision** | **Publication bias** | **Quality of the evidence** |
| --- | --- | --- | --- | --- | --- | --- | --- |
| VO2max | High | Serious concern ^a^ | Serious concern ^b^ | No serious concern | No serious concern | No serious concern | Low |
| VO2peak | High | No serious concern | Serious concern ^b^ | No serious concern | No serious concern | No serious concern | Moderate |
| SBP | High | Serious concern ^a^ | No serious concern | No serious concern | No serious concern | No serious concern | Moderate |
| DBP | High | Serious concern ^a^ | Serious concern ^b^ | No serious concern | No serious concern | No serious concern | Low |
| HRrest | High | No serious concern | Serious concern ^b^ | No serious concern | No serious concern | No serious concern | Moderate |
| HRmax | High | No serious concern | Serious concern ^b^ | No serious concern | Serious concern ^c^ | No serious concern | Low |

*Note: VO2max: maximal oxygen consumption, VO2peak: peak oxygen uptake, HRmax: max heart rate, HRrest: resting heart rate, SBP: systolic blood pressure, DBP: diastolic blood pressure*

*RCT, randomised controlled trial; GRADE, the Grading of Recommendations, Assessment, Development and Evaluations.*

*a: Downgraded one level due to some concerns about risk of bias*

*b: Downgraded one level due to unexplained heterogeneity*

*c: Downgraded one level due to wide confidence interval crossing the line of no effect*

eFigure 1. Risk of bias graph

Green plus = low risk of bias. Yellow question mark = some concerns for bias. Red minus = high risk of bias. Overall bias is equal to the highest level of bias of the remaining five sections.


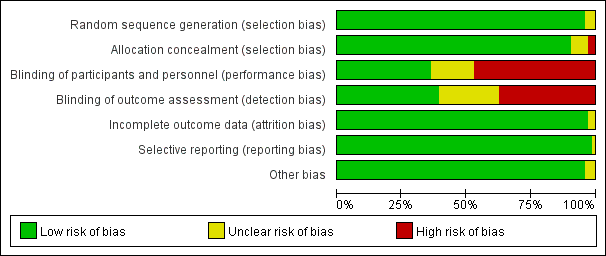


eFigure 2. Risk of bias summary


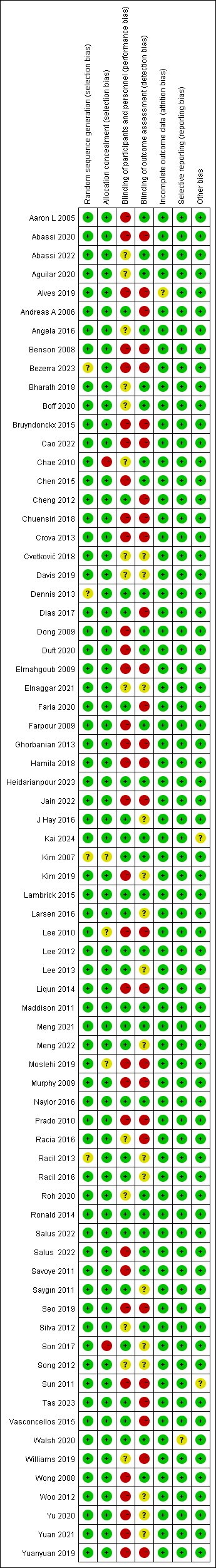


eFigure 3. Funnel plot

#### Funnel plot for maximal oxygen consumption (n=20 studies)


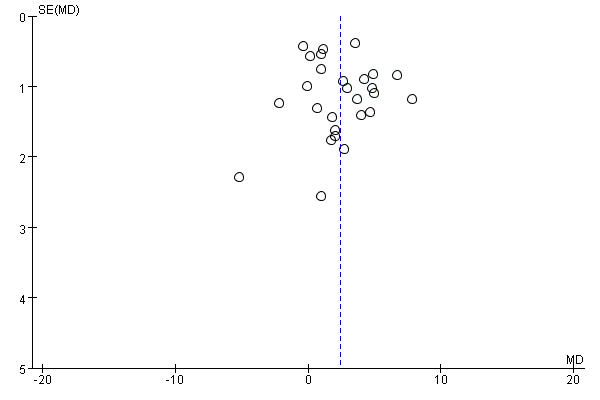


#### Funnel plot for Peak oxygen consumption (n=19 studies)


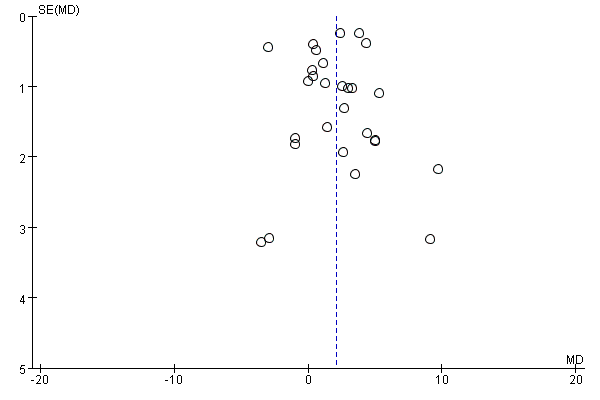


#### Funnel plot for systolic pressure (n=39 studies)


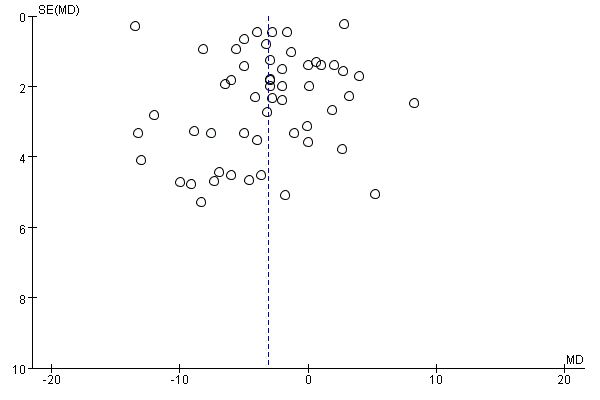


#### Funnel plot for diastolic pressure (n=34 studies)


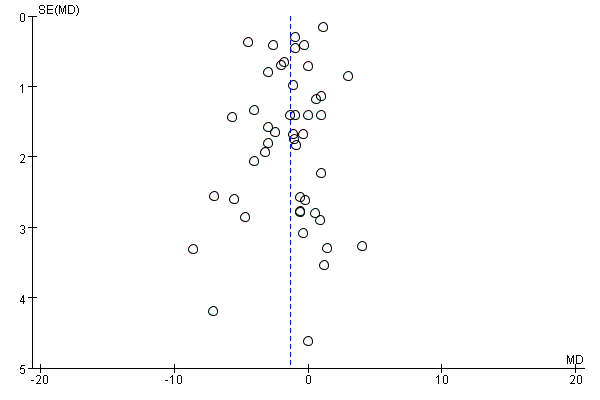


#### Funnel plot for Heart rate max (n=12 studies)


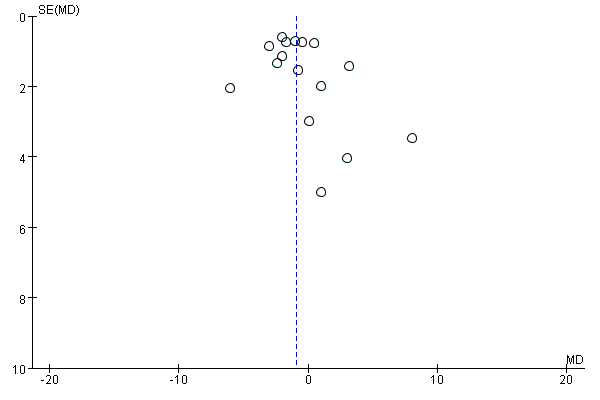


#### Funnel plot for Heart rate rest (n=11 studies)


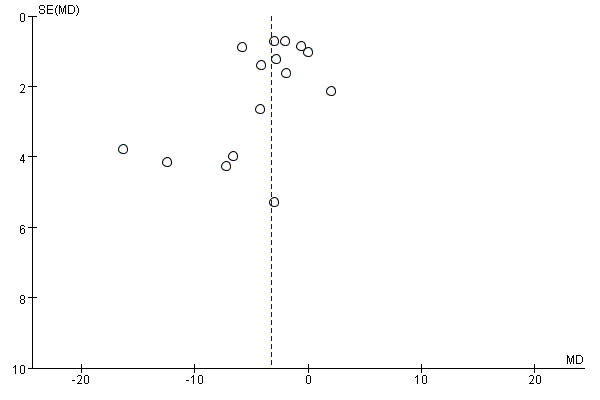


eFigure 4. Results of subgroup analysis of maximal oxygen consumption

#### Group by Exercise intensityMotion time

####
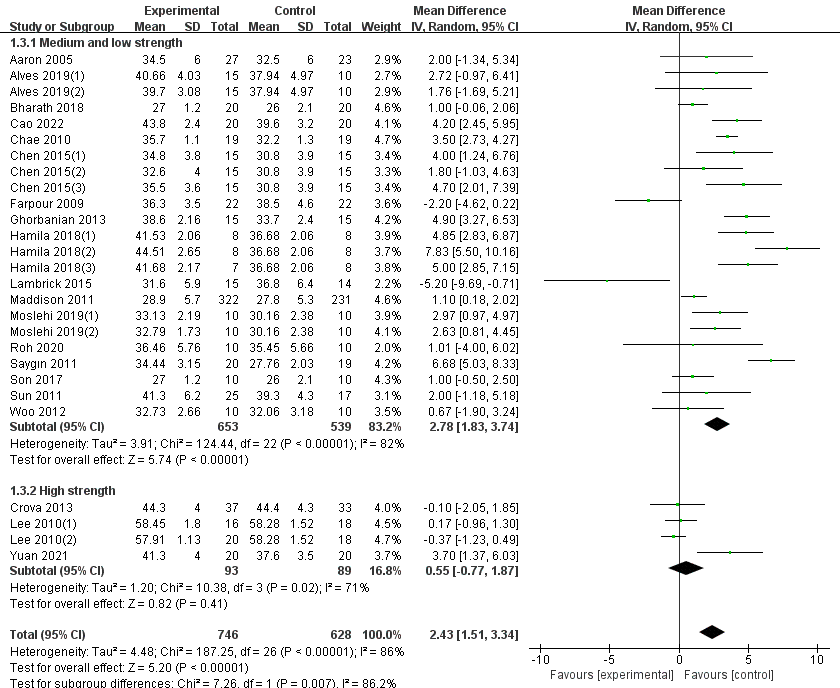


#### Group by Training volume (weeks)


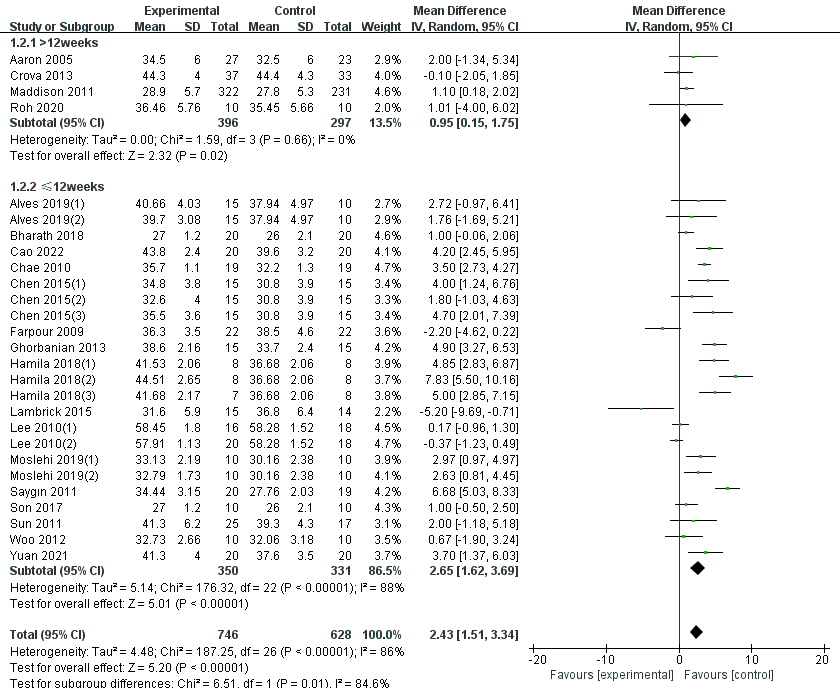


#### Group by motion frequency


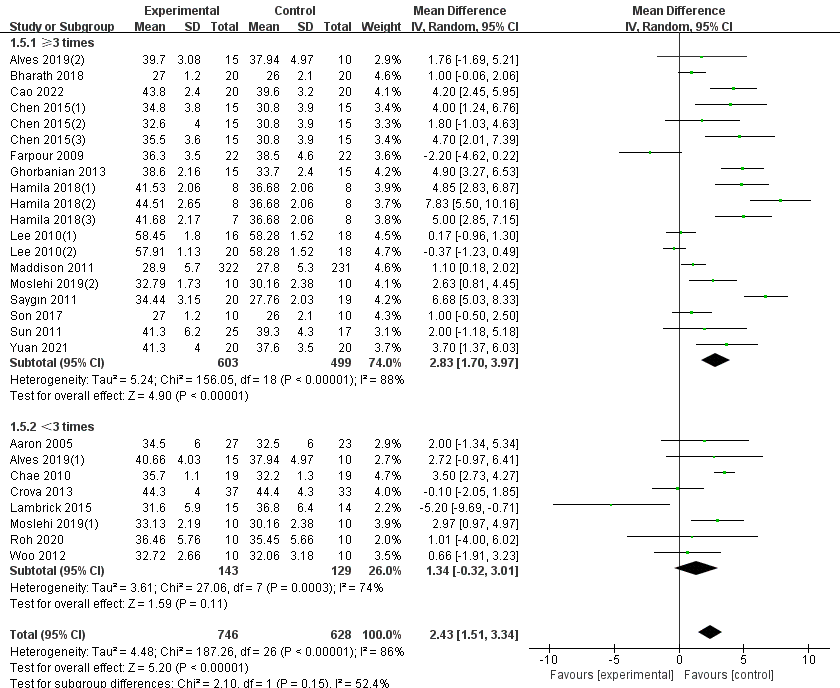


#### Group by Motion time

**
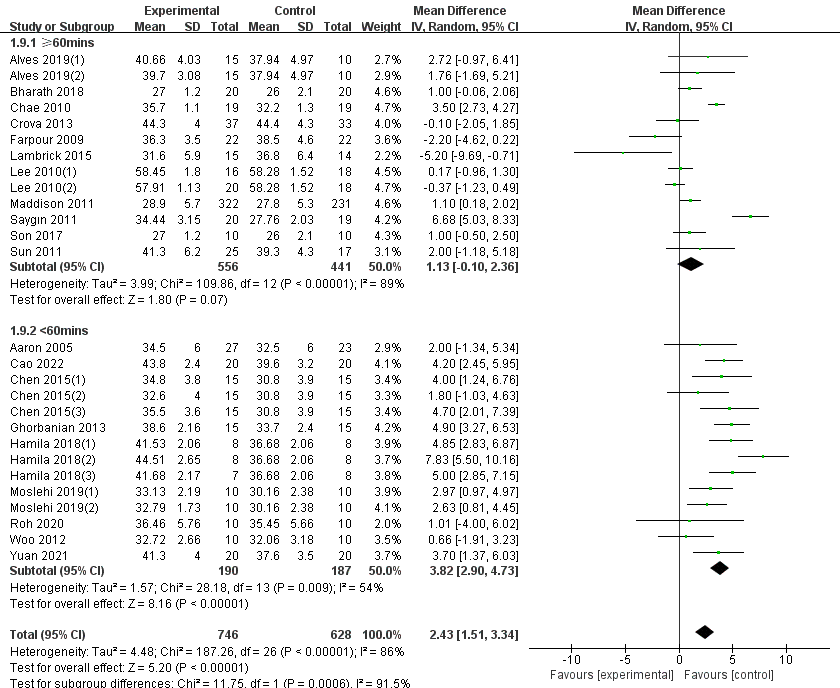
**

#### Group by Area

**
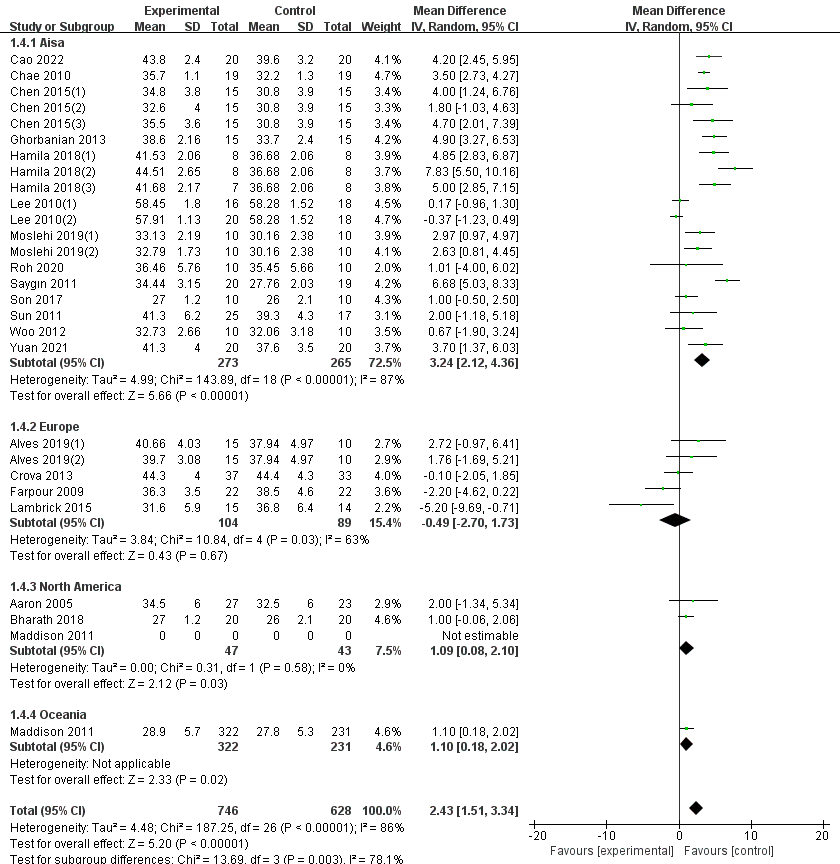
**

eFigure 5. Results of subgroup analysis of Peak oxygen consumption

#### Group by Exercise intensityMotion time

**
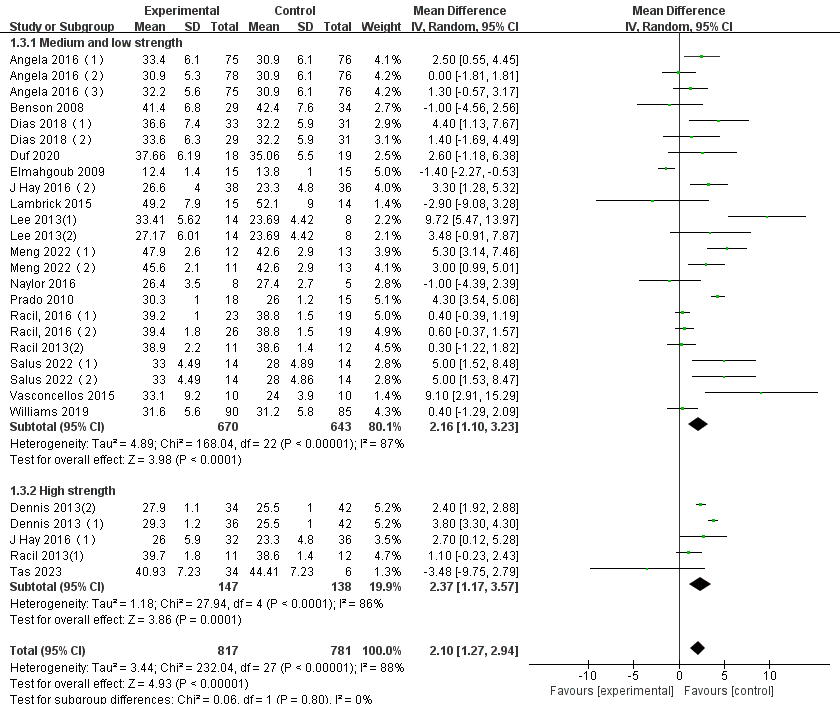
**

#### Group by Training volume (weeks)

**
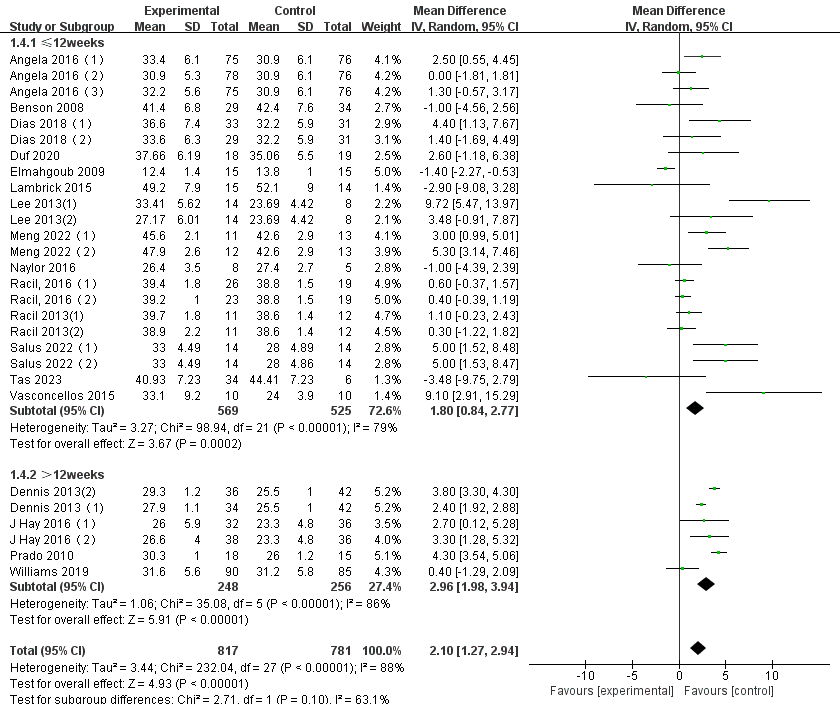
**

#### Group by motion frequency

**
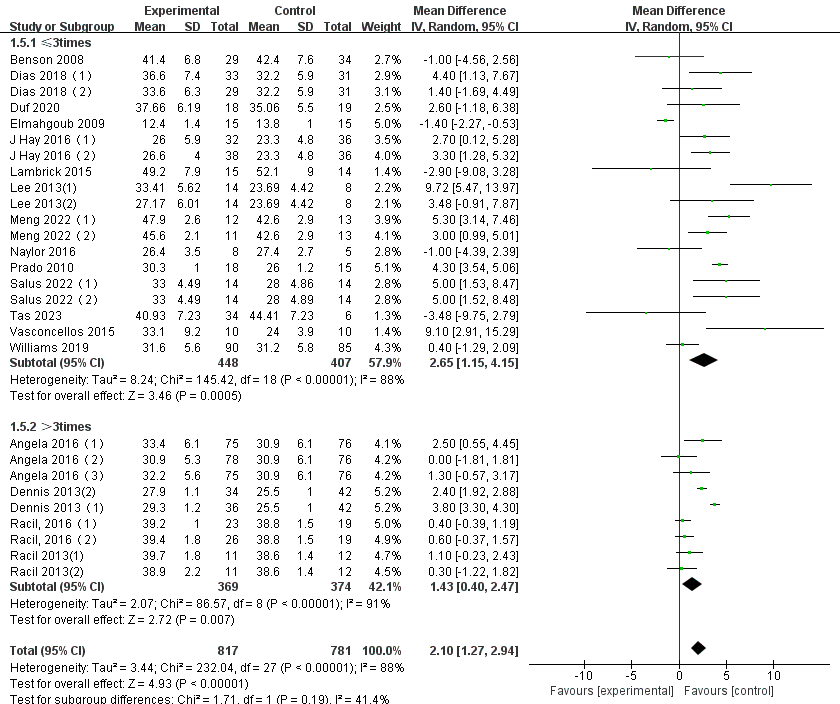
**

#### Group by Motion time

**
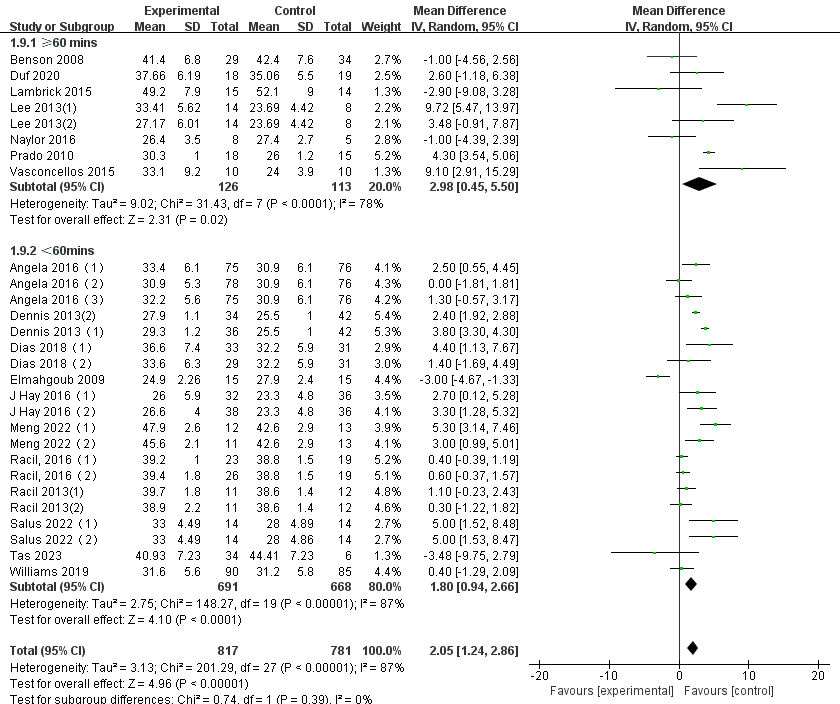
**

eFigure 6. Results of subgroup analysis of systolic pressure

#### Group by Exercise intensity

**
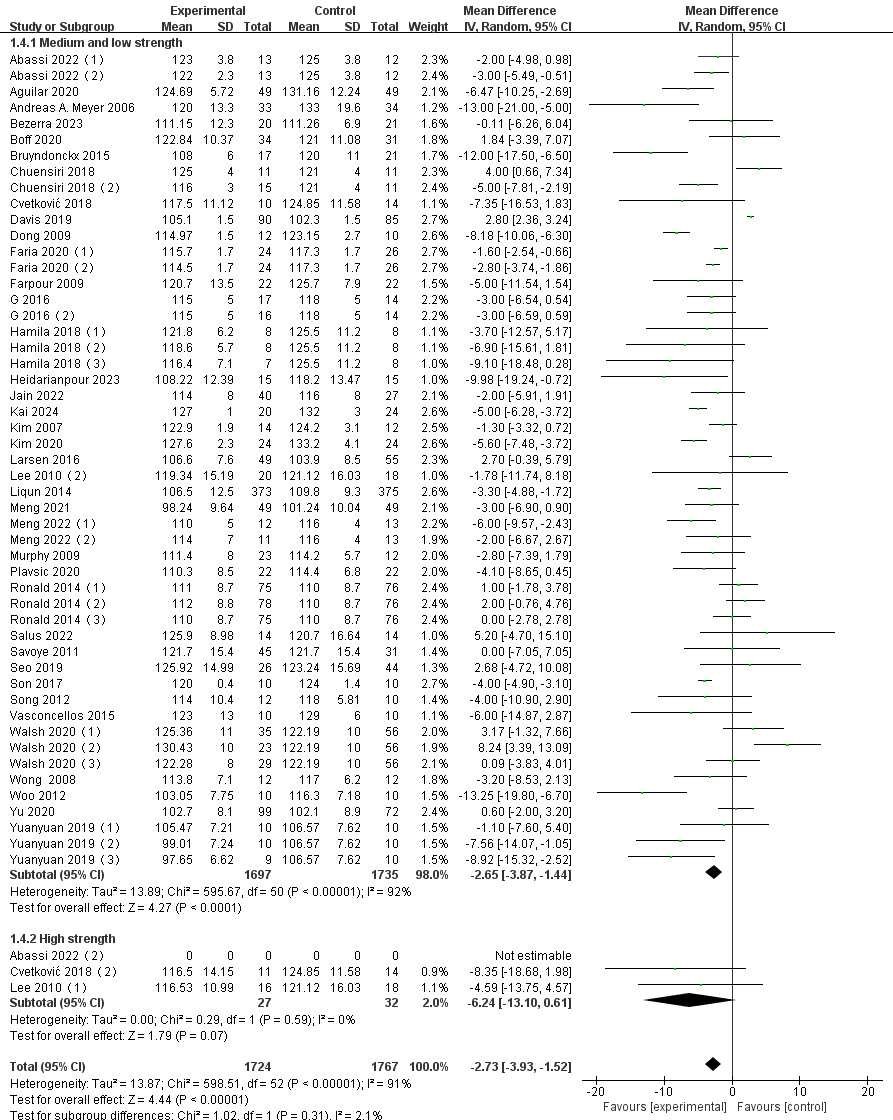
**

#### Group by Training volume (weeks)

**
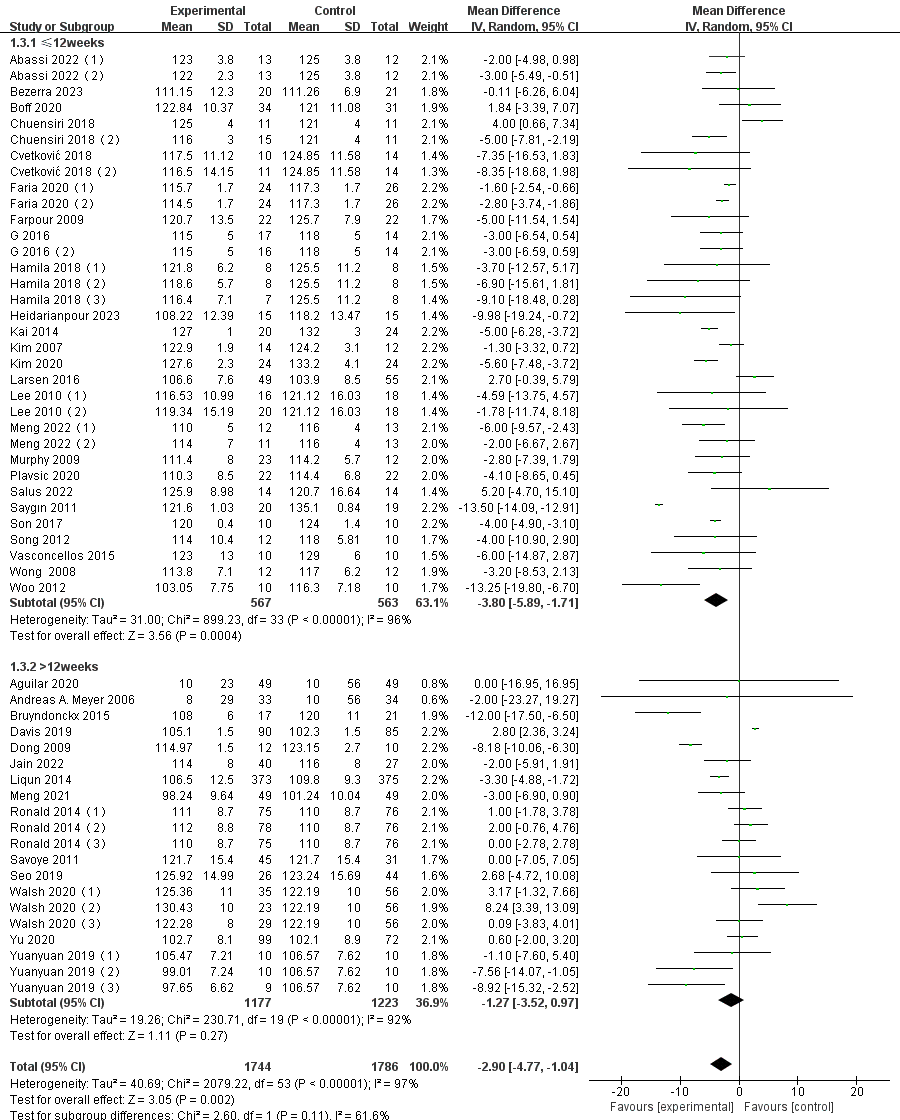
**

#### Group by motion frequency

**
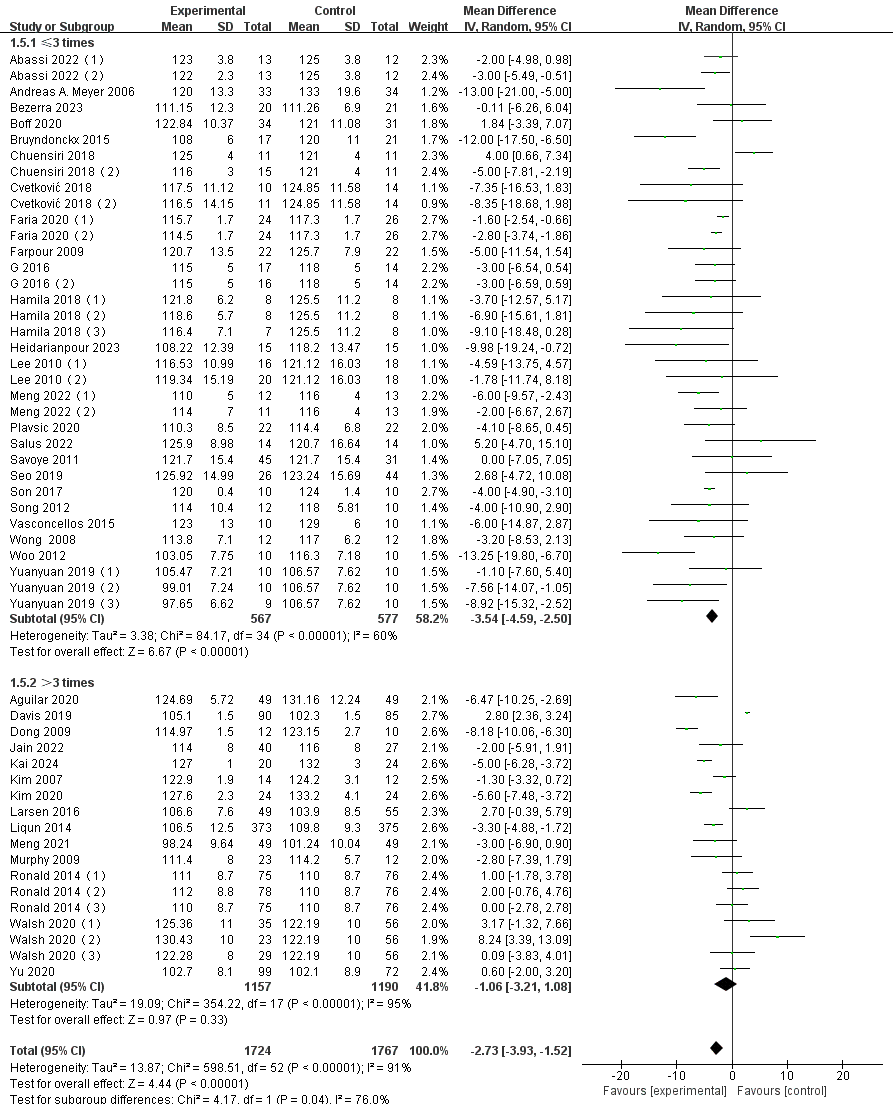
**

#### Group by Motion time

**
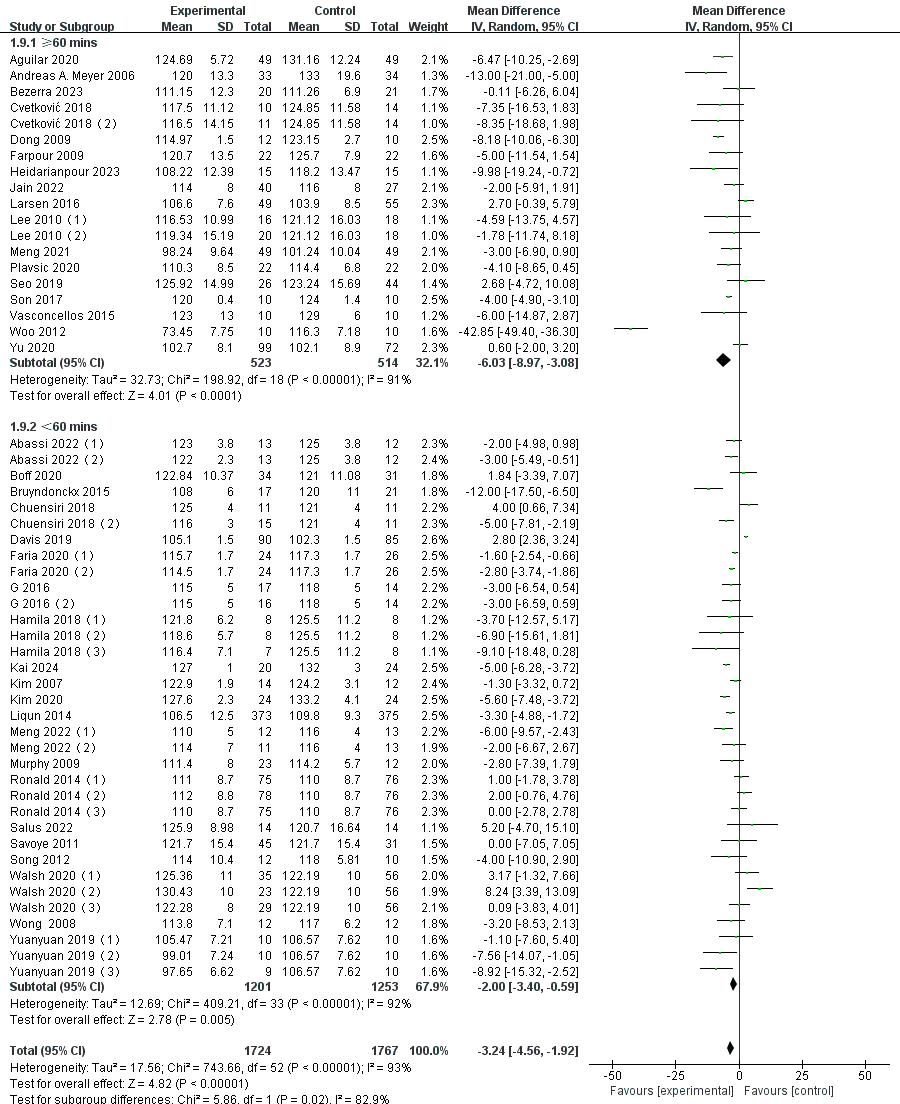
**

eFigure 7. Results of subgroup analysis of diastolic pressure

#### Group by Training volume (weeks)

**
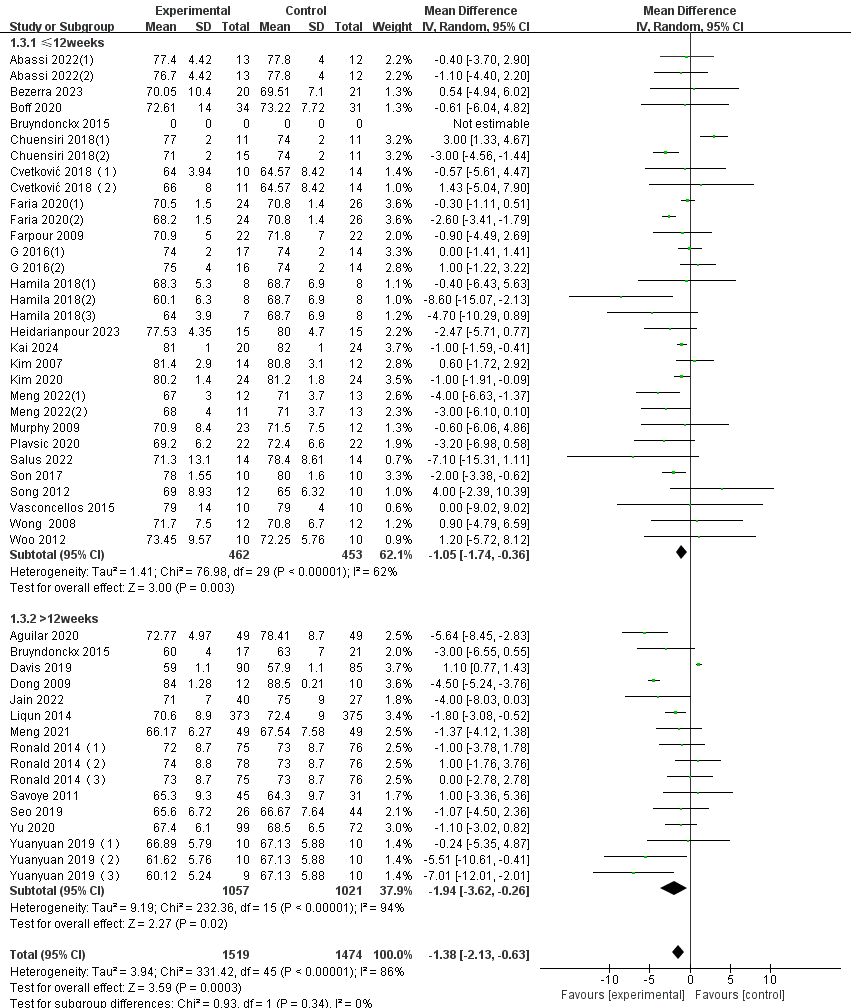
**

#### Group by motion frequency

**
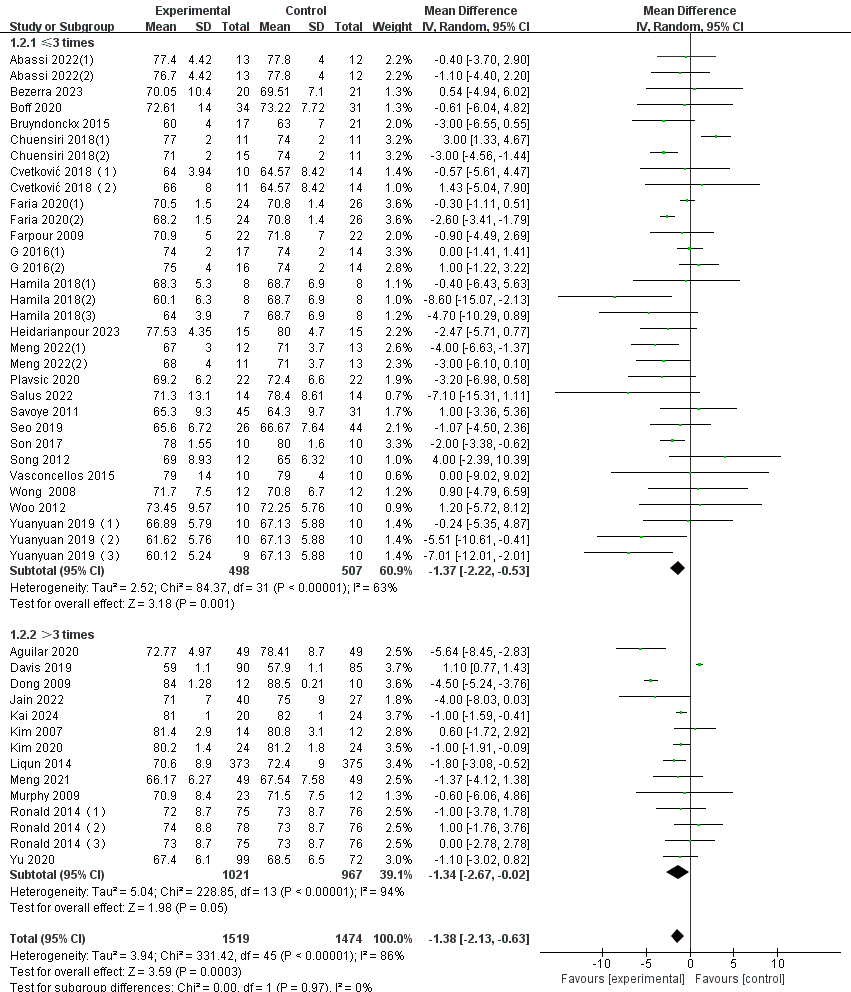
**

#### Group by motion time

**
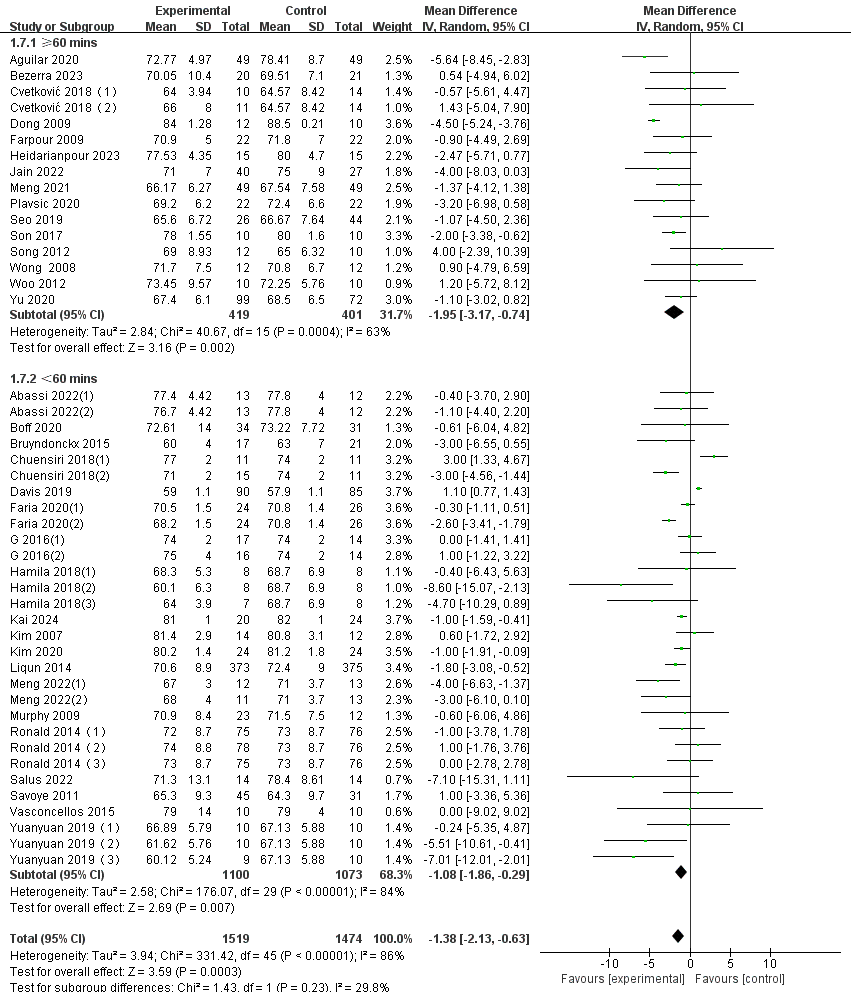
**

eFigure 8. Results of subgroup analysis of Heart rate max

#### Group by Training volume (weeks)


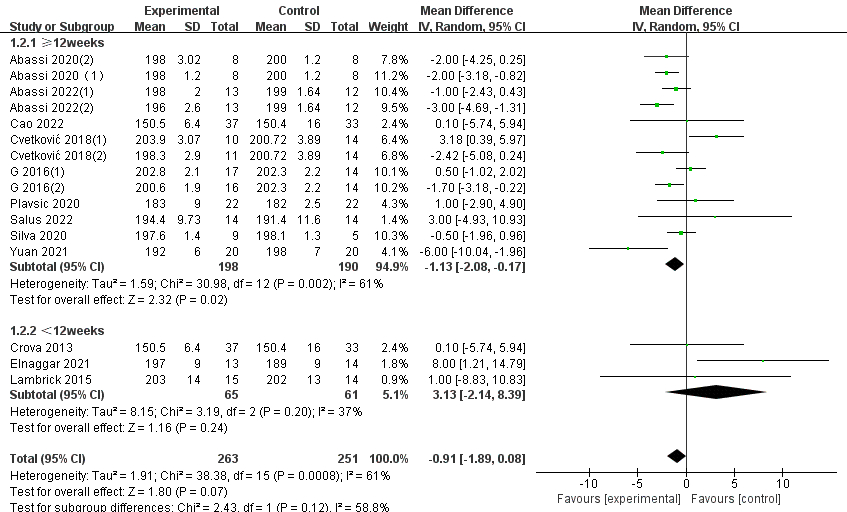


#### Group by motion frequency

**
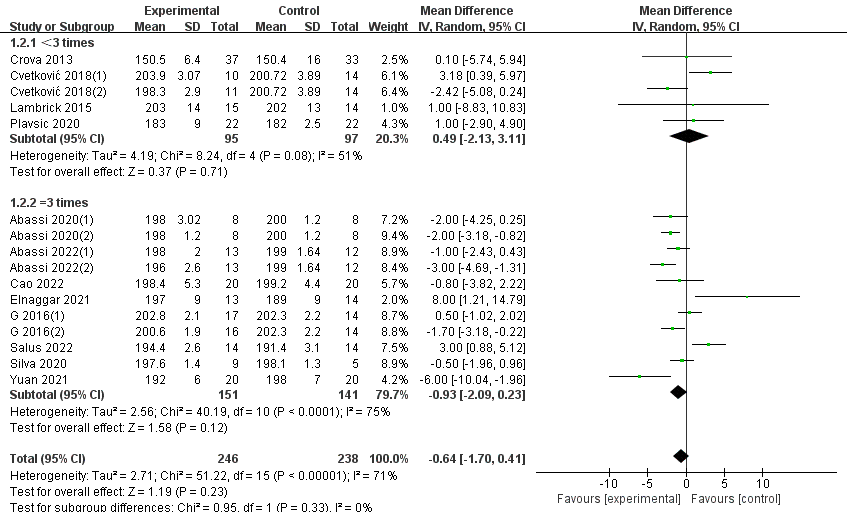
**

#### Group by motion time

**
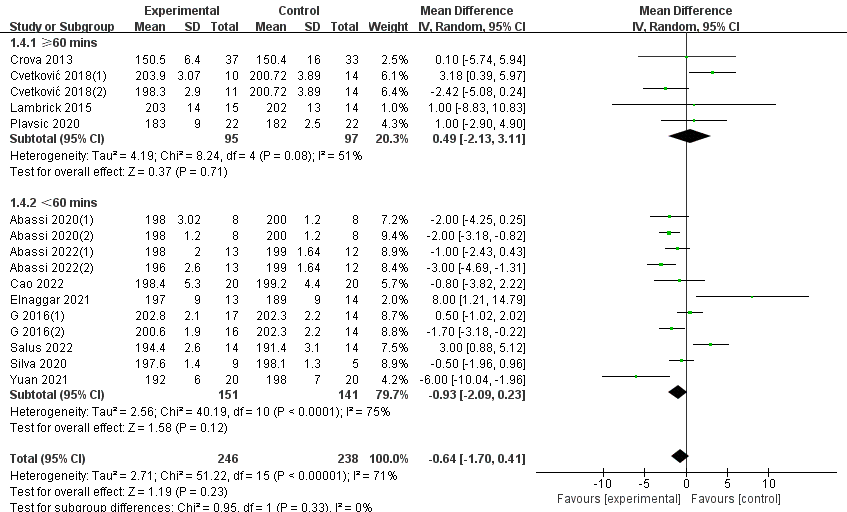
**

eFigure 9. Results of subgroup analysis of Heart rate rest

#### Group by Training volume (weeks)

**
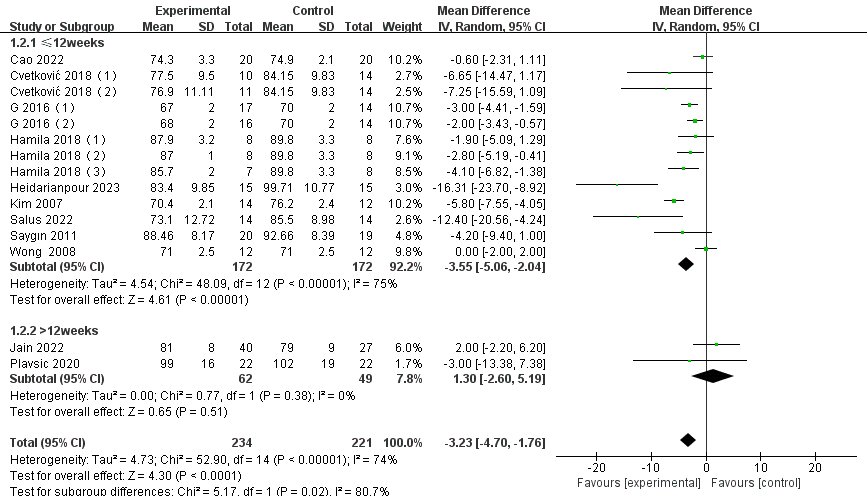
**

#### Group by motion frequency

**
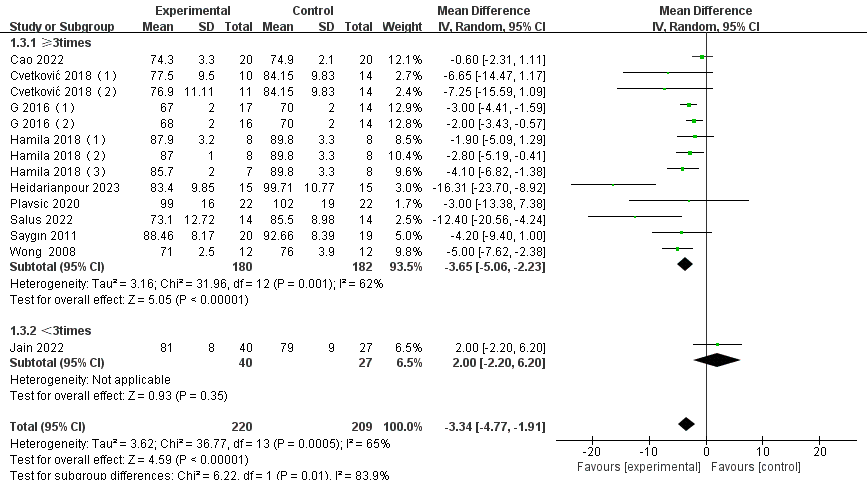
**

#### Group by motion time

**
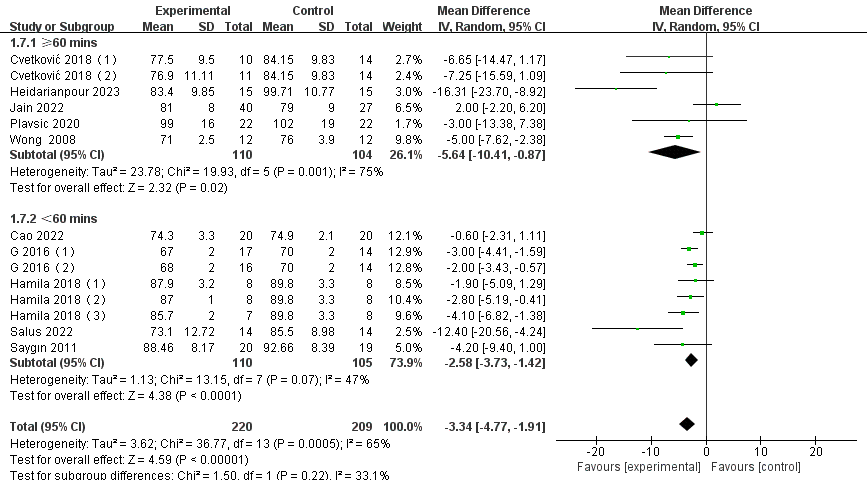
**

**References:**

**1.** Kaufman C, Kelly AS, Kaiser DR, Steinberger J, Dengel DR. Aerobic-exercise training improves ventilatory efficiency in overweight children. *Pediatr Exerc Sci.* 2007;19(1):82-92.

**2.** Logan NE, Occidental N, Watrous J, et al. The complex associations between adiposity, fitness, mental wellbeing and neurocognitive function after exercise: A randomized crossover trial in preadolescent children. *Prog Brain Res.* 2024;283:123-165.

**3.** Peña A, Olson ML, Ayers SL, et al. Inflammatory Mediators and Type 2 Diabetes Risk Factors before and in Response to Lifestyle Intervention among Latino Adolescents with Obesity. *Nutrients.* 2023;15(11).

**4.** Pott W, Albayrak O, Hebebrand J, Pauli-Pott U. Course of depressive symptoms in overweight youth participating in a lifestyle intervention: associations with weight reduction. *J Dev Behav Pediatr.* 2010;31(8):635-640.

**5.** Huang Z, Li J, Liu Y, Zhou Y. Effects of different exercise modalities and intensities on body composition in overweight and obese children and adolescents: a systematic review and network meta-analysis. *Front Physiol.* 2023;14:1193223.

**6.** Martin-Smith R, Cox A, Buchan DS, Baker JS, Grace F, Sculthorpe N. High Intensity Interval Training (HIIT) Improves Cardiorespiratory Fitness (CRF) in Healthy, Overweight and Obese Adolescents: A Systematic Review and Meta-Analysis of Controlled Studies. *Int J Environ Res Public Health.* 2020;17(8).

**7.** Wu J, Yang Y, Yu H, Li L, Chen Y, Sun Y. Comparative effectiveness of school-based exercise interventions on physical fitness in children and adolescents: a systematic review and network meta-analysis. *Front Public Health.* 2023;11:1194779.

**8.** Ribeiro B, Forte P, Vinhas R, et al. Reply to: Comment on: "The Benefits of Resistance Training in Obese Adolescents: A Systematic Review and Meta-analysis". *Sports Med Open.* 2023;9(1):12.

**9.** Wang Y, Wang S, Meng X, Zhou H. Effect of high-intensity interval training and moderate-intensity continuous training on cardiovascular risk factors in adolescents: Systematic review and meta-analysis of randomized controlled trials. *Physiol Behav.* 2024;275:114459.

**10.** Leinaar E, Alamian A, Wang L. A systematic review of the relationship between asthma, overweight, and the effects of physical activity in youth. *Ann Epidemiol.* 2016;26(7):504-510.

**11.** Oliveira A, Monteiro Â, Jácome C, Afreixo V, Marques A. Effects of group sports on health-related physical fitness of overweight youth: A systematic review and meta-analysis. *Scand J Med Sci Sports.* 2017;27(6):604-611.

**12.** Wang C, Tian Z, Hu Y, Luo Q. Physical activity interventions for cardiopulmonary fitness in obese children and adolescents: a systematic review and meta-analysis. *Bmc Pediatr.* 2023;23(1):558.

**13.** Martin-Smith R, Cox A, Buchan DS, Baker JS, Grace F, Sculthorpe N. High Intensity Interval Training (HIIT) Improves Cardiorespiratory Fitness (CRF) in Healthy, Overweight and Obese Adolescents: A Systematic Review and Meta-Analysis of Controlled Studies. *Int J Environ Res Public Health.* 2020;17(8).

**14.** Abassi W, Ouerghi N, Feki M, et al. Effects of moderate- vs. high-intensity interval training on physical fitness, enjoyment, and affective valence in overweight/obese female adolescents: a pre-/post-test study. *Eur Rev Med Pharmacol Sci.* 2023;27(9):3809-3822.

**15.** Haapala EA, Lubans DR, Jaakkola T, et al. Which indices of cardiorespiratory fitness are more strongly associated with brain health in children with overweight/obesity? *Scand J Med Sci Sports.* 2024;34(1):e14549.

**16.** Mora-Gonzalez J, Esteban-Cornejo I, Solis-Urra P, et al. The effects of an exercise intervention on neuroelectric activity and executive function in children with overweight/obesity: The ActiveBrains randomized controlled trial. *Scand J Med Sci Sports.* 2024;34(1):e14486.

**17.** Panagiotopoulos C, Ronsley R, Al-Dubayee M, et al. The centre for healthy weights--shapedown BC: a family-centered, multidisciplinary program that reduces weight gain in obese children over the short-term. *Int J Environ Res Public Health.* 2011;8(12):4662-4678.

**18.** Rodriguez-Ayllon M, Plaza-Florido A, Mendez-Gutierrez A, et al. The effects of a 20-week exercise program on blood-circulating biomarkers related to brain health in overweight or obese children: The ActiveBrains project. *J Sport Health Sci.* 2023;12(2):175-185.

**19.** Dai L, Xie B. Adaptations to Optimized Interval Training in Soccer Players: A Comparative Analysis of Standardized Methods for Individualizing Interval Interventions. *J Sports Sci Med.* 2023;22(4):760-768.

**20.** Bila WC, Romano M, Dos SL, et al. Body fat, cardiovascular risk factors and polymorphism in the FTO gene: randomized clinical trial and different physical exercise for adolescents. *J Pediatr (Rio J).* 2023;99(2):139-146.

**21.** Ben OO, Elloumi M, Zouhal H, et al. Effect of individualized exercise training combined with diet restriction on inflammatory markers and IGF-1/IGFBP-3 in obese children. *Ann Nutr Metab.* 2010;56(4):260-266.

**22.** Boer PH, Meeus M, Terblanche E, et al. The influence of sprint interval training on body composition, physical and metabolic fitness in adolescents and young adults with intellectual disability: a randomized controlled trial. *Clin Rehabil.* 2014;28(3):221-231.

**23.** Zhou L, Liang W, He Y, et al. A school-family blended multi-component physical activity program for Fundamental Motor Skills Promotion Program for Obese Children (FMSPPOC): protocol for a cluster randomized controlled trial. *Bmc Public Health.* 2023;23(1):369.

**24.** de Souza MD, Dos SML, de Souza MM, et al. Effects of 12 Weeks of Family and Individual Multi-Disciplinary Intervention in Overweight and Obese Adolescents under Cardiometabolic Risk Parameters: A Clinical Trial. *Int J Environ Res Public Health.* 2023;20(20).

**25.** Wu Q, Han R, Li Z, et al. Effect of virtual reality-based exercise and physical exercise on adolescents with overweight and obesity: study protocol for a randomised controlled trial. *Bmj Open.* 2023;13(10):e75332.

**26.** González-Gálvez N, Soler-Marín A, Abelleira-Lamela T, Abenza-Cano L, Mateo-Orcajada A, Vaquero-Cristóbal R. Eight weeks of high-intensity interval vs. sprint interval training effects on overweight and obese adolescents carried out during the cool-down period of physical education classes: randomized controlled trial. *Front Public Health.* 2024;12:1394328.

**27.** Williams TR, Walker RC, Dearing CG. Nurse facilitated 5000 m running at Parkrun improves vulnerable adolescent health in a high deprivation area: A matched pair randomized control trial. *Public Health Nurs.* 2024;41(3):458-465.

**28.** Carrel AL, Clark RR, Peterson SE, Nemeth BA, Sullivan J, Allen DB. Improvement of Fitness, Body Composition, and Insulin Sensitivity in Overweight Children in a School-Based Exercise Program: A Randomized, Controlled Study. *Archives of pediatrics & adolescent medicine.* 2005;159(10):963-968.

**29.** Meyer AA, Kundt G, Lenschow U, Schuff-Werner P, Kienast W. Improvement of Early Vascular Changes and Cardiovascular Risk Factors in Obese Children After a Six-Month Exercise Program. *J Am Coll Cardiol.* 2006;48(9):1865-1870.

**30.** Alberga AS, Prud Homme D, Sigal RJ, et al. Effects of aerobic training, resistance training, or both on cardiorespiratory and musculoskeletal fitness in adolescents with obesity: the HEARTY trial. *Applied physiology, nutrition, and metabolism.* 2016;41(3):255-265.

**31.** Alves ASR, Venâncio TL, Honório SAA, Martins JM. Multicomponent training with different frequencies on body composition and physical fitness in obese children. *Anais da Academia Brasileira de Ciências.* 2019;91(4).

**32.** Abassi W, Ouerghi N, Ghouili H, Haouami S, Bouassida A. Greater effects of high- compared with moderate-intensity interval training on thyroid hormones in overweight/obese adolescent girls. *Horm Mol Biol Clin Investig.* 2020;41(4).

**33.** Aguilar-Cordero MJ, Rodríguez-Blanque R, Leon-Ríos X, Expósito RM, García GI, Sánchez-López AM. Influence of Physical Activity on Blood Pressure in Children With Overweight/Obesity: A Randomized Clinical Trial. *Am J Hypertens.* 2020;33(2):131-136.

**34.** Abassi W, Ouerghi N, Nikolaidis PT, et al. Interval Training with Different Intensities in Overweight/Obese Adolescent Females. *Int J Sports Med.* 2022;43(5):434.

**35.** Singh ABMT. The effect of high-intensity progressive resistance training on adiposity in children: a randomized controlled trial. *Int J Obesity.* 2008;32(8):1016-1027.

**36.** Bruyndonckx L, Hoymans VY, De Guchtenaere A, et al. Diet, exercise, and endothelial function in obese adolescents. *Pediatrics (Evanston).* 2015;135(3):e653.

**37.** Bharath LP, Choi WW, Cho J, et al. Combined resistance and aerobic exercise training reduces insulin resistance and central adiposity in adolescent girls who are obese: randomized clinical trial. *Eur J Appl Physiol.* 2018;118(8):1653-1660.

**38.** Boff RDM, Dornelles MA, Feoli AMP, Gustavo ADS, Oliveira MDS. Transtheoretical model for change in obese adolescents: MERC randomized clinical trial. *J Health Psychol.* 2020;25(13-14):2272-2285.

**39.** Bezerra TA, Souza FA, Pessoa M, et al. Effects of a multicomponent intervention on cardiovascular risk factors in overweight children: a randomized clinical trial in light of complex systems. *Int J Environ Health Res.* 2023;33(12):1368-1378.

**40.** Chae HW, Kwon YN, Rhie YJ, et al. Effects of a structured exercise program on insulin resistance, inflammatory markers and physical fitness in obese Korean children. *J Pediatr Endocrinol Metab.* 2010;23(10):1065-1072.

**41.** Cheng Hongling PPZR. Effects of eight weeks exercise prescription intervention on aerobic capacity,boby composition,blood lipid and C-reactive protein in obese adolescents. *Journal of Jilin University (Medicine Edition).* 2012;38(04):745-749.

**42.** Crova C, Struzzolino I, Marchetti R, et al. Cognitively challenging physical activity benefits executive function in overweight children. *J Sports Sci.* 2014;32(3):201-211.

**43.** Chen Qiong CJZL. Effects of different exercise methods on body composition, inflammatory factors and exercise ability of obese adolescents. *Journal of Jilin University (Medicine Edition).* 2015;41(5):1070-1075.

**44.** Chuensiri N, Suksom D, Tanaka H. Effects of High-Intensity Intermittent Training on Vascular Function in Obese Preadolescent Boys. *Child Obes.* 2018;14(1):41-49.

**45.** Cvetković N, Stojanović E, Stojiljković N, Nikolić D, Scanlan AT, Milanović Z. Exercise training in overweight and obese children: Recreational football and high‐intensity interval training provide similar benefits to physical fitness. *Scand J Med Sci Spor.* 2018;28(S1):18-32.

**46.** Cao M, Tang Y, Zou Y. Integrating High-Intensity Interval Training into a School Setting Improve Body Composition, Cardiorespiratory Fitness and Physical Activity in Children with Obesity: A Randomized Controlled Trial. *J Clin Med.* 2022;11(18):5436.

**47.** Jun DG. Comprehensive in terventions affected blood pressureblood lipids of simple obesity of adolescen t during weight loss. *Journal of Shandong Institute of Physical Education and Sports.* 2009;25(12):42-48.

**48.** Dennis BA, Ergul A, Gower BA, Allison JD, Davis CL. Oxidative Stress and Cardiovascular Risk in Overweight Children in an Exercise Intervention Program. *Child Obes.* 2013;9(1):15-21.

**49.** Dias KA, Ingul CB, Tjønna AE, et al. Effect of High-Intensity Interval Training on Fitness, Fat Mass and Cardiometabolic Biomarkers in Children with Obesity: A Randomised Controlled Trial. *Sports Med.* 2017;48(3):733-746.

**50.** Davis CL, Litwin SE, Pollock NK, et al. Exercise effects on arterial stiffness and heart health in children with excess weight: The SMART RCT. *Int J Obesity.* 2020;44(5):1152-1163.

**51.** Duft RG, Castro A, Bonfante ILP, et al. Altered metabolomic profiling of overweight and obese adolescents after combined training is associated with reduced insulin resistance. *Sci Rep-Uk.* 2020;10(1).

**52.** Elmahgoub SM, Lambers S, Stegen S, Van Laethem C, Cambier D, Calders P. The influence of combined exercise training on indices of obesity, physical fitness and lipid profile in overweight and obese adolescents with mental retardation. *Eur J Pediatr.* 2009;168(11):1327-1333.

**53.** Elnaggar RK, Shendy MA, Elfakharany MS. Effect of 8 Weeks of Incremental Aerobic Training on Inflammatory Mediators, Cardiorespiratory Indices, and Functional Capacity in Obese Children With Bronchial Asthma. *Pediatr Exerc Sci.* 2021;33(1):23-31.

**54.** Farpour-Lambert NJ, Aggoun Y, Marchand LM, Martin XE, Herrmann FR, Beghetti M. Physical Activity Reduces Systemic Blood Pressure and Improves Early Markers of Atherosclerosis in Pre-Pubertal Obese Children. *J Am Coll Cardiol.* 2009;54(25):2396-2406.

**55.** Faria WF, Mendonça FR, Santos GC, Kennedy SG, Elias RGM, Stabelini Neto A. Effects of 2 Methods of Combined Training on Cardiometabolic Risk Factors in Adolescents: A Randomized Controlled Trial. *Pediatr Exerc Sci.* 2020;32(4):217-226.

**56.** Ghorbanian B, Ravassi A, Reza M, Hedayati M. The Effects of Rope Training on Lymphocyte ABCA1 Expression, Plasma ApoA-I and HDL-c in Boy Adolescents. *International Journal of Endocrinology and Metabolism.* 2013;11(2).

**57.** Hamila A, Younes M, Cottin F, et al. Effects of walking exercises on body composition, heart rate variability, and perceptual responses in overweight and obese adolescents. *Sci Sport.* 2018;33(5):e191-e202.

**58.** Heidarianpour A, Shokri E, Sadeghian E, Cheraghi F, Razavi Z. Combined training in addition to cortisol reduction can improve the mental health of girls with precocious puberty and obesity. *Front Pediatr.* 2023;11.

**59.** J Hay KWAM, Sellers HDER, P Gardiner JM. Physical activity intensity and type 2 diabetes risk in overweight

youth: A randomized trial. *Published ahead of advance online publication.* 2015;40(2):1-19.

**60.** Jain V, Kumar B, Sharma A, et al. A comprehensive yoga programme for weight reduction in children & adolescents with obesity: A randomized controlled trial. *Indian J Med Res.* 2022;155(3):387.

**61.** Kim ES, Im JA, Kim KC, et al. Improved insulin sensitivity and adiponectin level after exercise training in obese Korean youth. *Obesity (Silver Spring).* 2007;15(12):3023-3030.

**62.** Kim J, Son W, Headid III RJ, Pekas EJ, Noble JM, Park S. The effects of a 12-week jump rope exercise program on body composition, insulin sensitivity, and academic self-efficacy in obese adolescent girls. *Journal of Pediatric Endocrinology and Metabolism.* 2019;33(1):129-137.

**63.** Jianjiao WKXB. Effects of Combined Motion Intervention on Body Composition，Cardiovascular Risk Factors

and Cardiopulmonary Fitness of Obese Female Adolescents. *Chinese General Practice.* 2024;27(9):1109-1117.

**64.** Lee YH, Song YW, Kim HS, et al. The effects of an exercise program on anthropometric, metabolic, and cardiovascular parameters in obese children. *Korean Circ J.* 2010;40(4):179-184.

**65.** Lee S, Bacha F, Hannon T, Kuk JL, Boesch C, Arslanian S. Effects of Aerobic Versus Resistance Exercise Without Caloric Restriction on Abdominal Fat, Intrahepatic Lipid, and Insulin Sensitivity in Obese Adolescent Boys. *Diabetes.* 2012;61(11):2787-2795.

**66.** Lee S, Deldin AR, White D, et al. Aerobic exercise but not resistance exercise reduces intrahepatic lipid content and visceral fat and improves insulin sensitivity in obese adolescent girls: a randomized controlled trial. *American journal of physiology: endocrinology and metabolism.* 2013;305(10):E1222-E1229.

**67.** Zhao Liqun GHXK. Evaluation of the effect of comprehensive intervention and its

cost-benefit analysis in children with obesity. *Acta Nutrimenta Sinica.* 2014;36(5):426-429.

**68.** Lambrick D, Westrupp N, Kaufmann S, Stoner L, Faulkner J. The effectiveness of a high-intensity games intervention on improving indices of health in young children. *J Sports Sci.* 2015;34(3):190-198.

**69.** Larsen KT, Huang T, Ried-Larsen M, Andersen LB, Heidemann M, Møller NC. A Multi-Component Day-Camp Weight-Loss Program Is Effective in Reducing BMI in Children after One Year: A Randomized Controlled Trial. *Plos One.* 2016;11(6):e157182.

**70.** Murphy EC, Carson L, Neal W, Baylis C, Donley D, Yeater R. Effects of an exercise intervention using Dance Dance Revolution on endothelial function and other risk factors in overweight children. *Int J Pediatr Obes.* 2009;4(4):205-214.

**71.** Maddison R, Foley L, Ni Mhurchu C, et al. Effects of active video games on body composition: a randomized controlled trial. *The American Journal of Clinical Nutrition.* 2011;94(1):156-163.

**72.** Moslehi Ebrahim MZKB. Playing in form of outdoor aerobic exercise is more effective than indoor treadmill

exercise on serum Orexin-A and weight loss in obese adolescent boys. *Obesity Medicine.* 2019;22(4):1-17.

**73.** Zhaogang Meng ZZJH. Exercise nutrition intervention in adolescent obese patients. *Southwest Defense Medicine.* 2021;31(6).

**74.** Meng C, Yucheng T, Shu L, Yu Z. Effects of school-based high-intensity interval training on body composition, cardiorespiratory fitness and cardiometabolic markers in adolescent boys with obesity: a randomized controlled trial. *Bmc Pediatr.* 2022;22(1).

**75.** Naylor LH, Davis EA, Kalic RJ, et al. Exercise training improves vascular function in adolescents with type 2 diabetes. *Physiol Rep.* 2016;4(4).

**76.** Prado DM, Silva AG, Trombetta IC, et al. Exercise training associated with diet improves heart rate recovery and cardiac autonomic nervous system activity in obese children. *Int J Sports Med.* 2010;31(12):860-865.

**77.** Racil G, Ben OO, Hammouda O, et al. Effects of high vs. moderate exercise intensity during interval training on lipids and adiponectin levels in obese young females. *Eur J Appl Physiol.* 2013;113(10):2531-2540.

**78.** Ronald J Sigal ASAG. Effects of Aerobic Training, Resistance Training, or Both on Percentage Body Fat and Cardiometabolic Risk Markers in Obese Adolescents The Healthy Eating Aerobic and Resistance Training in Youth Randomized Clinical Trial. *Jama Pediatr.* 2014;168(11):1006-1014.

**79.** Racil G, Zouhal H, Elmontassar W, et al. Plyometric exercise combined with high-intensity interval training improves metabolic abnormalities in young obese females more so than interval training alone. *Appl Physiol Nutr Metab.* 2016;41(1):103-109.

**80.** Ghazi Racila HZWE. abnormalities in young obese females more so than interval training alone. *Applied Physiology, Nutrition and Metabolism.* 2016;102(5):1-26.

**81.** Roh H, Cho S, So W. Effects of Regular Taekwondo Intervention on Oxidative Stress Biomarkers and Myokines in Overweight and Obese Adolescents. *Int J Env Res Pub He.* 2020;17(7):2505.

**82.** Saygın O, Zcan. The effect of twelve week aerobic exercise programme on health related physical fitness components and blood lipids in obese girls. *African Journal of Pharmacy and Pharmacology.* 2011;5(12).

**83.** Sun MX, Huang XQ, Yan Y, et al. One-hour after-school exercise ameliorates central adiposity and lipids in overweight Chinese adolescents: a randomized controlled trial. *Chin Med J (Engl).* 2011;124(3):323-329.

**84.** Savoye M, Nowicka P, Shaw M, et al. Long-term results of an obesity program in an ethnically diverse pediatric population. *Pediatrics (Evanston).* 2011;127(3):402.

**85.** Kim HB, Stebbins CL, Chai JH, Song JK. Taekwondo training and fitness in female adolescents. *J Sports Sci.* 2011;29(2):133-138.

**86.** Silva DAS, Petroski EL, Pelegrini A, Guglielmo LGA. Effect of Physical Exercise on the Cardiorespiratory Response in Overweight Adolescents. *Turkish Journal of Endocrinology and Metabolism.* 2012;16(1):14-18.

**87.** Son WM, Sung KD, Bharath LP, Choi KJ, Park SY. Combined exercise training reduces blood pressure, arterial stiffness, and insulin resistance in obese prehypertensive adolescent girls. *Clin Exp Hypertens.* 2017;39(6):546-552.

**88.** Seo Y, Lim H, Kim Y, et al. The Effect of a Multidisciplinary Lifestyle Intervention on Obesity Status, Body Composition, Physical Fitness, and Cardiometabolic Risk Markers in Children and Adolescents with Obesity. *Nutrients.* 2019;11(1):137.

**89.** Salus M, Tillmann V, Remmel L, et al. Effect of Sprint Interval Training on Cardiometabolic Biomarkers and Adipokine Levels in Adolescent Boys with Obesity. *Int J Env Res Pub He.* 2022;19(19):12672.

**90.** Salus M, Tillmann V, Remmel L, et al. Effect of supervised sprint interval training on cardiorespiratory fitness and body composition in adolescent boys with obesity. *J Sport Sci.* 2022;40(18):2010-2017.

**91.** Tas E, Landes RD, Diaz EC, et al. Effects of short‐term supervised exercise training on liver fat in adolescents with obesity: a randomized controlled trial. *Obesity.* 2023;31(11):2740-2749.

**92.** Vasconcellos F, Seabra A, Cunha F, et al. Health markers in obese adolescents improved by a 12-week recreational soccer program: a randomised controlled trial. *J Sport Sci.* 2015;34(6):564-575.

**93.** Wong PC, Chia MY, Tsou IY, et al. Effects of a 12-week exercise training programme on aerobic fitness, body composition, blood lipids and C-reactive protein in adolescents with obesity. *Ann Acad Med Singap.* 2008;37(4):286-293.

**94.** Woo J, Shin KO, Yoo J, Park S, Kang S. The effects of detraining on blood adipokines and antioxidant enzyme in Korean overweight children. *Eur J Pediatr.* 2012;171(2):235-243.

**95.** Williams CF. Exercise effects on quality of life, mood, and self-worth in

overweight children: the SMART randomized controlled trialExercise effects on quality of life, mood, and self-worth in

overweight children: the SMART randomized controlled trial. 2019.

**96.** Walsh JJ, Bonafiglia JT, Goldfield GS, et al. Interindividual variability and individual responses to exercise training in adolescents with obesity. *Applied physiology, nutrition, and metabolism.* 2020;45(1):45-54.

**97.** Yuanyuan Qiao XW. Evaluation of Intervention Effect of Different Intensity Football Exercise on Body Composition and Blood Biochemical Indexes of Zhuang Obese Pupils. *Chin J Sch Health.* 2019;40(8):1238-1241.

**98.** Yu H, Li F, Hu Y, et al. Improving the Metabolic and Mental Health of Children with Obesity: A School-Based Nutrition Education and Physical Activity Intervention in Wuhan, China. *Nutrients.* 2020;12(1):194.

**99.** Lingling Y. Effects of high-intensity intermittent exercise on cardiopulmonary fitness, body composition and blood lipid level of overweight or obese male adolescents. *Chinese Journal of Physical Medicine and Rehabilitation.* 2021;43(3):251-253.
